# Supplementary material for: Mediating role of learned helplessness’ components in the association between health literacy/social support and self-management among maintenance haemodialysis patients in Changsha, China: a cross-sectional study
Source: BMJ Open. 2023 Aug 28;13(8):e068601. doi: 10.1136/bmjopen-2022-068601 (PMC10462950; doi:10.1136/bmjopen-2022-068601)
Supplement: Supplementary data [file bmjopen-2022-068601supp001.pdf]

Supplementary File 1

STROBE 2007 (v4) Statement—Checklist of items that should be included in reports of *cross-sectional studies*

Section/Topic	Item #	Recommendation	Reported on page #
**Title and abstract**	1	(*a*) Indicate the study's design with a commonly used term in the title or the abstract	P1
		(*b*) Provide in the abstract an informative and balanced summary of what was done and what was found	P1-2
**Introduction**			
Background/rationale	2	Explain the scientific background and rationale for the investigation being reported	P3-7
Objectives	3	State specific objectives, including any prespecified hypotheses	P8
**Methods**			
Study design	4	Present key elements of study design early in the paper	P8
Setting	5	Describe the setting, locations, and relevant dates, including periods of recruitment, exposure, follow-up, and data collection	P8-9
Participants	6	(*a*) Give the eligibility criteria, and the sources and methods of selection of participants	P8-9
Variables	7	Clearly define all outcomes, exposures, predictors, potential confounders, and effect modifiers. Give diagnostic criteria, if applicable	P9-11
Data sources/ measurement	8*	For each variable of interest, give sources of data and details of methods of assessment (measurement). Describe comparability of assessment methods if there is more than one group	P9-11
Bias	9	Describe any efforts to address potential sources of bias	P11
Study size	10	Explain how the study size was arrived at	P11
Quantitative variables	11	Explain how quantitative variables were handled in the analyses. If applicable, describe which groupings were chosen and why	P11
Statistical methods	12	(*a*) Describe all statistical methods, including those used to control for confounding	P13
		(*b*) Describe any methods used to examine subgroups and interactions	P12-13
		(*c*) Explain how missing data were addressed	P12
		(*d*) If applicable, describe analytical methods taking account of sampling strategy	P9
		(*e*) Describe any sensitivity analyses	N/A

**Results**			
Participants	13*	(a) Report numbers of individuals at each stage of study—eg numbers potentially eligible, examined for eligibility, confirmed eligible, included in the study, completing follow-up, and analysed	N/A
		(b) Give reasons for non-participation at each stage	N/A
		(c) Consider use of a flow diagram	N/A
Descriptive data	14*	(a) Give characteristics of study participants (eg demographic, clinical, social) and information on exposures and potential confounders	P13-14
		(b) Indicate number of participants with missing data for each variable of interest	N/A
Outcome data	15*	Report numbers of outcome events or summary measures	P15
Main results	16	(*a*) Give unadjusted estimates and, if applicable, confounder-adjusted estimates and their precision (eg, 95% confidence interval). Make clear which confounders were adjusted for and why they were included	P13
		(*b*) Report category boundaries when continuous variables were categorized	N/A
		(*c*) If relevant, consider translating estimates of relative risk into absolute risk for a meaningful time period	N/A
Other analyses	17	Report other analyses done—eg analyses of subgroups and interactions, and sensitivity analyses	N/A
**Discussion**			
Key results	18	Summarise key results with reference to study objectives	P18
Limitations	19	Discuss limitations of the study, taking into account sources of potential bias or imprecision. Discuss both direction and magnitude of any potential bias	P23
Interpretation	20	Give a cautious overall interpretation of results considering objectives, limitations, multiplicity of analyses, results from similar studies, and other relevant evidence	P18-22
Generalisability	21	Discuss the generalisability (external validity) of the study results	P23
**Other information**			
Funding	22	Give the source of funding and the role of the funders for the present study and, if applicable, for the original study on which the present article is based	P24

*Give information separately for cases and controls in case-control studies and, if applicable, for exposed and unexposed groups in cohort and cross-sectional studies.

Note: An Explanation and Elaboration article discusses each checklist item and gives methodological background and published examples of transparent reporting. The STROBE checklist is best used in conjunction with this article (freely available on the Web sites of PLoS Medicine at http://www.plosmedicine.org/, Annals of Internal Medicine at http://www.annals.org/, and Epidemiology at http://www.epidem.com/). Information on the STROBE Initiative is available at www.strobe-statement.org.
