# Supplementary figures and images for: Mediating role of learned helplessness’ components in the association between health literacy/social support and self-management among maintenance haemodialysis patients in Changsha, China: a cross-sectional study
Source: BMJ Open. 2023 Aug 28;13(8):e068601. doi: 10.1136/bmjopen-2022-068601 (PMC10462950; doi:10.1136/bmjopen-2022-068601)

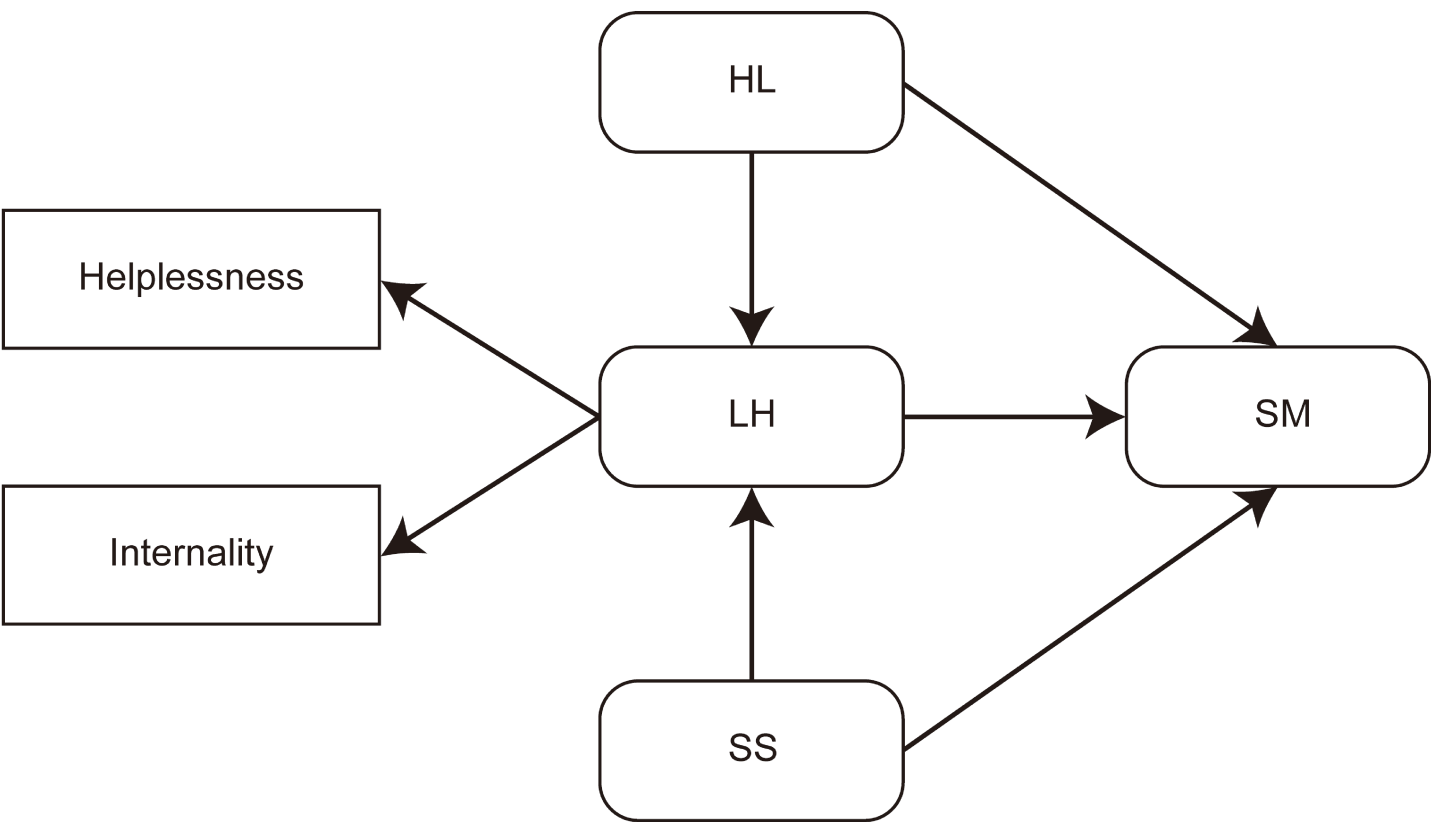

Supplement: Supplementary data [file bmjopen-2022-068601supp002.pdf]
