# Supplementary material for: Mediating role of learned helplessness’ components in the association between health literacy/social support and self-management among maintenance haemodialysis patients in Changsha, China: a cross-sectional study
Source: BMJ Open. 2023 Aug 28;13(8):e068601. doi: 10.1136/bmjopen-2022-068601 (PMC10462950; doi:10.1136/bmjopen-2022-068601)
Supplement: Author's manuscript [file bmjopen-2022-068601.draft_revisions.pdf]

BMJ Open is committed to open peer review. As part of this commitment we make the peer review history of every article we publish publicly available.

When an article is published we post the peer reviewers' comments and the authors' responses online. We also post the versions of the paper that were used during peer review. These are the versions that the peer review comments apply to.

The versions of the paper that follow are the versions that were submitted during the peer review process. They are not the versions of record or the final published versions. They should not be cited or distributed as the published version of this manuscript.

BMJ Open is an open access journal and the full, final, typeset and author-corrected version of record of the manuscript is available on our site with no access controls, subscription charges or pay-per-view fees (http://bmjopen.bmj.com).

If you have any questions on BMJ Open's open peer review process please email info.bmjopen@bmj.com

BMJ Open

The mediating role of learned helplessness's components in the association between health literacy/social support and self-management: a cross-sectional study

Journal:	*BMJ Open*
Manuscript ID	bmjopen-2022-068601
Article Type:	Original research
Date Submitted by the Author:	28-Sep-2022

Complete List of Authors:	Xie, Chunyan; Central South University, Xiangya Nursing School<br>Li, Li; The Second Xiangya Hospital of Central South University, Clinical Nursing Teaching and Research Section<br>Li, Ya-Min; The Second Xiangya Hospital of Central South University, Clinical Nursing Teaching and Research Section

Keywords:	MENTAL HEALTH, Dialysis < NEPHROLOGY, Change management < HEALTH SERVICES ADMINISTRATION & MANAGEMENT

SCHOLARONE™
Manuscripts

I, the Submitting Author has the right to grant and does grant on behalf of all authors of the Work (as defined in the below author licence), an exclusive licence and/or a non-exclusive licence for contributions from authors who are: i) UK Crown employees; ii) where BMJ has agreed a CC-BY licence shall apply, and/or iii) in accordance with the terms applicable for US Federal Government officers or employees acting as part of their official duties; on a worldwide, perpetual, irrevocable, royalty-free basis to BMJ Publishing Group Ltd ("BMJ") its licensees and where the relevant Journal is co-owned by BMJ to the co-owners of the Journal, to publish the Work in this journal and any other BMJ products and to exploit all rights, as set out in our licence.

The Submitting Author accepts and understands that any supply made under these terms is made by BMJ to the Submitting Author unless you are acting as an employee on behalf of your employer or a postgraduate student of an affiliated institution which is paying any applicable article publishing charge ("APC") for Open Access articles. Where the Submitting Author wishes to make the Work available on an Open Access basis (and intends to pay the relevant APC), the terms of reuse of such Open Access shall be governed by a Creative Commons licence – details of these licences and which Creative Commons licence will apply to this Work are set out in our licence referred to above.

Other than as permitted in any relevant BMJ Author's Self Archiving Policies, I confirm this Work has not been accepted for publication elsewhere, is not being considered for publication elsewhere and does not duplicate material already published. I confirm all authors consent to publication of this Work and authorise the granting of this licence.

For peer review only

The mediating role of learned helplessness's components in the association between health literacy/social support and self-management: a cross-sectional study

Chunyan Xie[1,2], Li Li[1–3], Yamin Li[1]

[1] Clinical Nursing Teaching and Research Section, The Second Xiangya Hospital, Central South University, Changsha, Hunan, 410011, People's Republic of China;

[2] XiangYa Nursing School, Central South University, Changsha, Hunan, 410013, People's Republic of China;

[3] The First Affiliated Hospital of Xinjiang Medical University, Urumqi, Xinjiang, 830011, People's Republic of China

Correspondence: Yamin Li, The Second Xiangya Hospital, Central South University, Changsha, Hunan, 410011, People's Republic of China, Email aminny@csu.edu.cn

For peer review only

Abstract

Objectives: To explored the multiple mediating roles of learned helplessness' core system in the relationship between health literacy/social support and self-management.

Design: Cross-sectional survey design.

Setting: Changsha, China.

Participants: 239 Chinese maintenance hemodialysis (MHD) patients.

For peer review only - http://bmjopen.bmj.com/site/about/guidelines.xhtml

Methods: Two multiple mediator models were constructed based on the COM-B model. 239 Chinese maintenance hemodialysis (MHD) patients participated in a cross-sectional study, which included surveys on the Learned Helplessness Scale for MHD patients, Dialysis Knowledge Questionnaire, Social Support Scale, and Self-Management Scale for Hemodialysis. The PROCESS macro in the SPSS program was used for mediated effects analysis.

Results: Helplessness and internality partially mediated the relationship between health literacy/social support and self-management [(β = -0.217, $P < 0.01$; β = 0.209, $P < 0.01$) / (β = -0.34, $P < 0.001$; β = 0.352, $P < 0.001$)]. The mediation effect size was 0.736 (95% CI [0.3287, 1.1694]) in the health literacy model, accounting for 43.05% of the total effect, and 0.292 (95% CI [0.2156, 0.3813]) in the social support model, accounting for 58.23% of the total effect. The differences in effect sizes for helplessness and internality in two models were -0.0383 (95% CI [-0.3195, 0.2483]), -0.0352 (95% CI [-0.1216, 0.0506]), respectively.

Conclusion: Health literacy/social support directly affects the MHD patients' self-management and indirectly affects it by changing the learned helplessness, such as increasing internality while reducing helplessness.

Keywords: learned helplessness, self-management, health literacy, social support, multiple mediations

Strengths and limitations of this study:

1.The study further explained the mechanism of health literacy/social support influences self-management.

For peer review only - http://bmjopen.bmj.com/site/about/guidelines.xhtml

2.It reveals the importance of learned helplessness on self-management behavior change in MHD patients.

3.Based on the ethnic diversity of China, participants could be recruited from a more diverse range of cultures and backgrounds.

4.Causality cannot be inferred from the current study, and future longitudinal studies are needed.

5.The self-reported data may pose a potential risk to the validity of the measurements.

BACKGROUND

As of 2017, there are approximately 700 million patients with Chronic Kidney Diseases (CKD) worldwide, with nearly one-third of them concentrated in China and India (132 million and 115 million, respectively) [1]. CKD is divided into five stages based on the glomerular filtration rate. CKD's fifth stage is called End Stage Kidney Disease (ESKD), when the patient's renal function has irreversibly declined or wholly lost. The main treatments to maintain ESKD patients' life are hemodialysis, peritoneal dialysis, and renal transplantation. Maintenance hemodialysis (MHD) is the most widely used alternative treatment in clinical practice[2 3]. MHD is defined as regular hemodialysis patients receive for more than three months with a frequency of 2-3 times per week[4]. In China, about more than 90% of ESKD patients are treated with MHD, and the annual growth rate is 20%[5].

Although MHD can prolong their lives, it cannot completely replace renal function. Patients must also slow their disease progression through self-management

For peer review only

such as long-term medications, restricted diet, fluid intake, regular exercise, and arteriovenous fistula care[6 7]. MHD patients' self-management means they are proactive in controlling their diet, performing self-care, and managing the disease with their caregivers to prevent complications and achieve desired life goals[8]. Curtin et al.[8] showed that self-management behaviors positively impacted MHD patients' overall health status. However, numerous studies pointed to the generally unsatisfactory self-management status in MHD patients. For example, less than 40% of them can strictly follow the doctor's advice to control their diet and water[9]. The overall level of Chinese MHD patients' self-management is low, with only 20.4% having a high level[10]. Effective implementation of self-management requires a combination of multiple factors. Therefore, it has become a priority for healthcare staff to investigate the controllable factors influencing MHD patients' self-management to improve their health status and quality of life.

Health literacy/social support and self-management

A growing body of researchers have focused on how to improve MHD patients' self-management behaviors. Health literacy is an important influencing factor for improving self-management behaviors[11], which reflects the degree of patient's awareness of the disease and determines their motivation and ability to acquire, understand, and use knowledge to improve their health[12]. Notably, nursing education aims to improve patients' perceptions of disease and overall health literacy to induce changes in their behaviors[13]. When patients are more aware of their disease and health,

For peer review only - http://bmjopen.bmj.com/site/about/guidelines.xhtml

they become aware of the importance of self-management and are more likely to adopt healthy behaviors, as confirmed by more studies. For example, Devraj et al.[14] showed that health literacy in CKD patients was positively associated with self-management, and a randomized controlled trial in Japan confirmed similar results[11]. Wu et al.[15] found that health literacy could influence CKD patients' self-management through the mediating role of self-efficacy, which is consistent with Hu et al.[16].

Second, social support is a factor that significantly impacts treating most patients with chronic diseases[17]. It is a multidimensional concept that refers to the spiritual and material support given to individuals by various parties such as family, relatives, friends, and social organizations[18]. Adequate social support can reduce the MHD patients' financial or psychological burdens and enable them to manage life better[19]. Several studies[20 21] have shown that the lack of family support in MHD patients is followed by poor medication adherence. Song et al.[22] used a structural equation modeling approach to confirm that social support directly affects MHD patients' self-management and, together with the sense of consistency, explained 69% of the variance in self-management behavior. Been-Dahmen et al.[23] noted through qualitative interviews that family, friends, professionals, and colleagues also provide essential emotional support to kidney transplant patients. Thus, social support is crucial for MHD patients to achieve their health goals.

Although previous researches have confirmed the relationship between health literacy, social support, and self-management, most studies have overlooked possible

For peer review only - http://bmjopen.bmj.com/site/about/guidelines.xhtml

potential pathways for this relationship. Therefore, the study was dedicated to exploring the multiple potential possibilities between health literacy/social support and self-management.

Learned helplessness

Learned helplessness (LH) has been described as a negative psychological state[24 25], whereby individuals feel they have little control over the adverse events and respond passively to the problems they encounter. Learned helplessness is often used to explain the phenomenon of diminished or absent motivation in individuals and extends to health-related problems[26]. For example, helpless patients adhere significantly less to regular treatment and seem to have more difficulty carrying out physicians' recommendations for diet, water, and exercise[27 28].

It has been suggested that learned helplessness is associated with high social support and health literacy. First, adequate social support gives patients more tangible resources for life and intangible resources for spirituality, which can buffer the multiple negative emotions associated with illness[29]. Those with high social support have access to disease-related knowledge through multiple channels to reduce helplessness when coping with the disease. Notably, family support always plays a vital role in reducing patients' learned helplessness[30]. Smallheer et al.[31] suggested that emotional support from family and friends, access to disease-related knowledge, and trust and support with fellow patients and healthcare staff can help prevent patients' helplessness. In addition, it has been suggested that social support and health literacy

For peer review only - http://bmjopen.bmj.com/site/about/guidelines.xhtml

can help maintain the harmony of physical and mental, and influence helplessness by integrating them[32].

Theoretical basis

The Capability, Opportunity, and Motivation - Behavior (COM-B) model is a theoretical model related to individual behavior change proposed by Michie[33], which can help us to comprehensively understand the barriers and facilitators in behavior interventions. COM-B model explains capability, opportunity, and motivation can affect individual behavior, among which capability and opportunity can, directly and indirectly, affect an individual's behavior through motivation.[34]. This study takes social support as an opportunity factor, health literacy as a capability factor, and self-management as the dependent variable. We explored the mediating role of learned helplessness's two core system as a motivation factor in the relationship between health literacy/social support and self-management in MHD patients, respectively, as shown in Figure 1.

The current study

There may be a relationship between health literacy/social support, learned helplessness, and self-management behaviors. However, the underlying mechanisms between these relationships remain to be further identified. In this study, we developed two multiple mediator models based on the COM-B model to help us understand the role of learned helplessness in forming and changing health behaviors. To test this, we made the following hypotheses.

For peer review only - http://bmjopen.bmj.com/site/about/guidelines.xhtml

Hypothesis 1 (H1): Health literacy/social support can influence two dimensions of learned helplessness (helplessness, internality).

Hypothesis 2 (H2): Two dimensions of learned helplessness (helplessness, internality) predict their self-management.

Hypothesis 3 (H3): Two dimensions of learned helplessness (helplessness, internality) parallelly mediate the relationship between health literacy/social support and self-management.

METHODS

Study design and participants

This is a cross-sectional study to investigate the association between health literacy/social support and self-management through the mediating role of leaned helplessness's component. We used the convenience sampling to select patients from a comprehensive Grade 3A hospital in Changsha, Hunan Province, China, who met the following criteria: (i) being treated with hemodialysis for ≥ 3 months, (ii) being informed and agreeing to participate in this study, and (iii) being conscious, being literate and having communication abilities. We excluded patients with mental illness, cognitive dysfunction, or serious complications. The purpose, significance of this study were clarified to the participants before the survey to obtain informed consent. The questionnaires were administered 1 hour after the start or 1 hour before the end of dialysis because the patients' blood circulation was more stable. Each participant took approximately 10-15 minutes to complete the questionnaire.

For peer review only - http://bmjopen.bmj.com/site/about/guidelines.xhtml

Measures

Health Literacy

We used the Dialysis Knowledge Questionnaire developed by Curtin[8] to measure the MHD patients' health literacy. The questionnaire was divided into five dimensions after cross-cultural adaptation by Li[35]. It includes anemia, diet and medication, kidney function, dialysis, and rehabilitation, with 24 items. The patients were asked to judge whether the items' descriptions were true, scoring 1 for a correct answer and 0 for an incorrect/ unknown answer. The total score was 24. The higher the score, the higher the patient's knowledge of dialysis. The questionnaire's content validity index (CVI) was 0.94, and the Cronbach's alpha coefficient was 0.701.

Social support

The Social Support Rating Scale (SSRS) developed by Xiao[36] was selected for this study. The scale consists of ten items involving three dimensions: objective support, subjective support, and support utilization. Items 1-4 and 8-10 were scored on a 4-point Likert scale. Item 5 has five subitems, each of which has four options from "none" to "fully support," scoring 1-4 points, respectively. The scores of items 6 and 7 are calculated according to the number of support sources. The total score ranges from 12 to 66, with higher scores indicating higher levels of social support. The Cronbach's alpha coefficient for the total scale was 0.896. This instrument has been used in Chinese renal dialysis patients.

For peer review only

For peer review only - http://bmjopen.bmj.com/site/about/guidelines.xhtml

Self-management

The Self-Management Scale for Hemodialysis (SMSH), developed by Song[37] in 2009, was used to measure MHD patients' self-management behaviors in this study. It has 20 items and was divided into four dimensions: problem-solving, performing self-care, partnership, and emotional processing. The scale was scored on a 4-point Likert scale, with scores 1-4 representing the frequency of the behavior, i.e., never, occasionally, often, and always, respectively. The total score was 20-80. The higher the score, the better the patient's self-management behavior. It was revised by Chinese Li[38], and its Cronbach's alpha coefficient was 0.813.

Learned helplessness

There is no special scale for MHD patients' LH. Considering this limitation, we formed a tool called LHS-MHD based on Nicassio's AHI[39] scale. Under his authorization, we conducted cross-cultural adaptation, psychological measurement, and evaluation. LHS-MHD has 11 items and was classified into two dimensions (helplessness/internality) based on the results of exploratory factor analysis (EFA) and confirmatory factor analysis (CFA), in line with the Learned Helplessness Theory. It used a five-point Likert scale with response options ranging from 1 = strongly disagree to 5 = strongly agree. Six items in internality are reverse scored. The higher the total score, the more severe the learned helplessness. In our previous study, the

For peer review only - http://bmjopen.bmj.com/site/about/guidelines.xhtml

scale showed a good internal consistency [(Cronbach's α of 0.759), retest reliability (n=30, intragroup correlation=0.772), and split-half reliability [0.774].

Statistical analysis

IBM SPSS version 25 was used to complete the descriptive statistics and correlation analysis of all variables. The PROCESS macro in SPSS, developed by Hayes (2013), was used for multiple mediation analyses. We adopted a bootstrap method (sampling was repeated 5000 times) to construct a 95% confidence interval for significance testing of mediating effects.

Patient and public involvement

Patients and/or the public were not involved in the design, or conduct, or reporting, or dissemination plans of this research.

Ethical consideration

The study was approved by the Ethics Committee of Xiangya Nursing School of Central South University on April 20, 2022 (No. E202255). Informed consent was obtained from all participants.

RESULTS

Demographic characteristics

For peer review only - http://bmjopen.bmj.com/site/about/guidelines.xhtml

The study population consisted of 239 Chinese MHD patients aged 21-86 with a mean age of 55.61 and a standard deviation (SD) = 13.31. Among them, 59.8% were male. The characteristics of the participants are shown in Table 1.

Table 1 Descriptive statistics of socialdemographic characters (n=239)

Variable		n	%
Gender			
	Male	143	59.8
	Female	96	40.2
Age (years)			
	18-44	46	19.2
	45-59	100	41.8
	≥60	93	38.9
Education level			
	Elementary school	26	10.9
	Middle school	126	52.7
	University and above	87	36.4
Employed status			
	On duty	10	4.2
	Part-time job	37	15.5
	Off duty	192	80.3
Protopathy			
	Hypertension	52	21.8
	Diabetes	48	20.1
	Chronic nephritis	105	43.9
	Polycystic kidney	20	8.4
	Others	14	5.9
Duration Of the MHD（years）			
	＜1	54	22.6
	1-3	42	17.6
	3-7	72	30.1
	≥ 7	71	29.7

Preliminary analysis

The correlations between the variables are listed in Table 2. The results of the correlation analyses were consistent with the hypotheses. All of them were

For peer review only - http://bmjopen.bmj.com/site/about/guidelines.xhtml

statistically significant at the P<0.05 level (two-tailed). First, social support, health literacy, and self-management were all negatively correlated with helplessness (r = -0.361, -0.599, and -0.463). Second, internality was positively associated with social support, health literacy, and self-management, with correlation coefficients of 0.445, 0.691, and 0.489, respectively. Third, social support and health literacy positively predicted self-management (r = 0.48, 0.636).

Table 2 Correlation coefficients of variables (n=239)

Variable	1	2	3	4	5
1.Helplessness	1				
2.Internality	-.140*	1			
3.Social Support	-.361**	.445**	1		
4.Self-Management	-.463**	.489**	.480**	1	
5.Health Literacy	-.599**	.691**	.443**	.636**	1

Note: *p＜0.05， ** p < 0.01

Parallel multiple mediator models

This study used three regression models to examine the parallel mediators' role of helplessness and internality in the relationship between health literacy/social support and self-management, respectively (Table 3 and Figure 2). Model 1, with health literacy/social support as the independent variable and helplessness as the dependent variable, showed that health literacy/social support was negatively associated with helplessness (β = -0.599, P < 0.001; β = -0.361, P < 0.001). In model 2, health

For peer review only - http://bmjopen.bmj.com/site/about/guidelines.xhtml

literacy/social support remained the independent variable, while internality was the dependent variable. The results showed significant regression coefficients ($\beta = 0.691$, $P < 0.001$; $\beta = 0.445$, $P < 0.001$). Model 3 used health literacy/social support as the independent variable, helplessness, internality as mediating variables, and self-management as the dependent variable. We found that in the health literacy model, the effects of both helplessness and internality on self-management were significant ($\beta = -0.217$, $P < 0.01$; $\beta = 0.209$, $P < 0.01$), and health literacy remained a significant positive predictor of self-management ($\beta = 0.362$, $P < 0.001$). Similarly, in the social support model, social support, helplessness, and internality all had significant effects on self-management ($\beta = 0.201$, $P < 0.001$; $\beta = -0.34$, $P < 0.001$; $\beta = 0.352$, $P < 0.001$). Therefore, we deduced that helplessness and internality partially mediate the relationship between health literacy/social support and self-management.

Table 3 Results of the multiple mediation analysis

Regression Model		Fitting Indices		Regression Coefficient	
**Outcome Variable**	**Predictor Variable**	**R^2**	**F**	**β**	**t**
Helplessness	Social Support	0.13	35.43	-0.36	-5.95***
Internality	Social Support	0.20	58.62	0.45	7.66***
	Social Support			0.20	3.43***
Self-management	Helplessness	0.43	58.29	-0.34	-6.44***
	Internality			0.35	6.38***
Helplessness	Health Literacy	0.36	132.60	-0.60	-11.52***

For peer review only - http://bmjopen.bmj.com/site/about/guidelines.xhtml

Internality	Health Literacy	0.48	216.54	0.69	14.72***
	Health Literacy			0.36	3.80***
Self-management	Helplessness	0.43	59.78	-0.22	-3.11**
	Internality			0.21	2.70**

*Note: *p < 0.05, **p < 0.01, ***p < 0.001.*

To confirm the significance of the model's total, direct, and indirect effects, we used a Bootstrap analysis with 5000 repeated sampling tests, and the confidence interval was set at 95%. The results showed significant parallel mediating effects of helplessness and internality on the relationship between health literacy/social support and self-management. The indirect effect size of health literacy via helplessness and internality on self-management was 0.736 (95% CI [0.3287, 1.1694]), accounting for 43.05% of the total effect. The indirect effect size of social support via helplessness and internality on self-management was 0.292 (95% CI [0.2156, 0.3813]), accounting for 58.23% of the total effect. In addition, the differences in effect sizes for helplessness and internality in the two models were -0.0383 (95% CI [-0.3195, 0.2483]), -0.0352 (95% CI [-0.1216, 0.0506]), respectively. It indicated that helplessness and internality did not differ significantly in the mediated effect size of the health literacy/social support and self-management relationship model. (Table 4)

Table 4 Bootstrap analysis of multiple mediation effects

	Effect Size	SE	LLCI	ULCI
X1 (Social Support)				

For peer review only - http://bmjopen.bmj.com/site/about/guidelines.xhtml

Total	0.50	0.06	0.38	0.62
Direct	0.21	0.06	0.09	0.33
Total Indirect	0.29	0.04	0.22	0.38
Indirect effect (M1=Helplessness)	0.13	0.03	0.08	0.19
Indirect effect (M2=Internality)	0.16	0.03	0.11	0.23
(C1)	-0.04	0.04	-0.12	0.05
X2 (Health Literacy)				
Total	1.71	0.13	1.44	1.97
Direct	0.97	.26	0.47	1.48
Total Indirect	0.74	0.21	0.33	1.17
Indirect effect (M1=Helplessness)	0.35	0.12	0.14	0.59
Indirect effect (M2=Internality)	0.39	0.14	0.11	0.68
(C1)	-0.04	0.14	-0.32	0.25

Note: C1=Helplessness minus Internality.

DISCUSSION

Psychological factors play a role in patients' responses to self-management skills[40], and the Learned Helplessness Theory has particular relevance in providing insight into these psychological factors. Numerous studies have found that learned helplessness is a common psychological experience for most patients with chronic diseases[31 41-43]. The severity, permanent, and irreversibility of ESKD dictate that a high risk of failure accompanies self-management in MHD patients. Patients who experience cycles of hope and dashed hopes are likely to induce learned helplessness. Previous scholars have proposed that health literacy and social support can improve

For peer review only

For peer review only - http://bmjopen.bmj.com/site/about/guidelines.xhtml

the MHD patients' self-management behavior. In this paper, we examine the mechanisms of health literacy/social support and self-management in MHD patients from the perspective of learned helplessness based on the COM-B model. The COM-B model suggests that behavior sources include motivation, capability, and opportunity. In this study, we used health literacy as a capability factor, social support as an opportunity factor, learned helplessness as a motivational factor, and self-management as a dependent variable. We constructed two multiple mediation models with different independent variables. Learned helplessness was divided into two core components. Helplessness is the individual's perception that their behavior cannot impact the outcome[44]. Internality originates from Rotter's[45] theory of psychological sources of control. It refers to the individual's perception that they believe that they are the most dominant force dominating their life. The results of the study confirm that for MHD patients, health literacy/social support can influence self-management through the indirect pathway of learned helplessness's two components (helplessness and internality).

Relevance of learned helplessness to self-management

According to Seligman, learned helplessness is essential to human behavior[46]. The present study first verified that helplessness is a negative predictor of self-management behavior, a result similar to other studies[47-49]. Helplessness plays an important cognitive, motivational, and behavioral function in individuals. It can influence patients' attitudes toward illness and treatment and determine their

For peer review only - http://bmjopen.bmj.com/site/about/guidelines.xhtml

health-related behaviors and persistence[50]. MHD patients feel isolated due to worsening kidney function, complications, and death anxiety. In addition, anxiety, panic, depression, regret, and social isolation are all feelings that contribute to the emotional burden of MHD patients. Many accept their condition with a sense of helplessness-a feeling that whatever they do will not have an impact on the future. An overwhelming helplessness may weaken patients' motivation to engage in treatment. This sense of helplessness may also be reinforced by recurrent experiences of uncontrollable symptoms, which may be deeply rooted in the background of living with the situation.

Next, we verified that the internality is a positive predictor of self-management, which is consistent with other researches[51 52]. Numerous scholars have argued that control is one of the basic psychological needs of humans, determined by humans' social attributes. Internality determines the degree to which an individual can grasp stressful events. When patients have a high internality, they can actively use various resources and establish a more positive way of coping with their illness. In this study, MHD patients with higher internality tend to perceive the existing treatment outcome as a result of their behavior. For example, for adverse events in disease treatment, patients more often attributed them to their lack of effort and believed they could mitigate the effects of adverse events by changing their behaviors.

The mediating role of learned helplessness

For peer review only - http://bmjopen.bmj.com/site/about/guidelines.xhtml

The results showed that health literacy/social support could influence the self-management of MHD patients through the mediating role of helplessness and internality, similar to the other studies[50 53]. First, health literacy reflects patients' perceptions of illness and treatment and can determine their ability and motivation to take positive steps to improve their health. Patients with higher health literacy have a higher ability to access medical information and communicate with medical staff and are better able to make rational judgments independently when faced with changes in their health status, thereby reducing feelings of helplessness. Individuals with a correct understanding of the disease can influence their emotional responses. When MHD patients have more knowledge about kidney disease and health care, their ability to perceive disease deterioration is enhanced, and their sense of control over the disease increases. Based on it, patients can more actively and purposefully engage in their health behavior.

Secondly, as an essential external resource for individuals, social support can effectively help buffer the harm caused by stressors and provide tangible and intangible support. Most MHD patients have experienced failures in disease control due to the irreversibility of ESKD, when the lack of social support, such as family support and medical support, can induce helplessness[54]. Helpless patients may feel they cannot change the status through their behaviors. So they have less internality and gradually show external control tendencies. They tend to attribute the outcome of their behaviors to external conditions (fate, social background, power, etc.) and believe that the result of events is more controlled by external conditions or the others

For peer review only - http://bmjopen.bmj.com/site/about/guidelines.xhtml

forces. They have less control over their behaviors. Most patients' behaviors are characterized by self-abandonment and lack of subjective initiatives, such as social avoidance, reluctance to chat, lack of novelty, and even decreased trust in people around them. Internality as an important motivational factor, is combined with social support to make patients feel the favorable internal and external environment and resources. The confidence to control the disease outcome is subsequently increased, which can motivate patients to take the initiative to adopt health-related behaviors.

Implications for future research

The results of this study confirmed that helplessness and internality are important ways for health literacy/social support to influence self-management behaviors. The results help us understand the mechanisms of possible changes in health-related behaviors of MHD patients and provide theoretical support for future studies. In the future, health care providers can focus on learned helplessness as an intervention target and identify and assess the level of learned helplessness as early as possible while providing health education and medical support for MHD patients. Patients can be empowered by encouraging or rewarding their self-management behaviors to improve their internality and reduce helplessness.

In addition, Seligman et al.[55] showed that individual helplessness also triggers a group's helplessness, harming individual behavior. Most MHD patients in China have wardmate groups, and learned helplessness events among peers may impact other

For peer review only - http://bmjopen.bmj.com/site/about/guidelines.xhtml

patients' behaviors. Future studies may investigate the interaction of learned helplessness in group on MHD patients' behaviors.

Limitations and future directions

The current study has several limitations that could be addressed in future research. First, based on the ethnic diversity of China, participants could be recruited from a more diverse range of cultures and backgrounds. Second, causality cannot be inferred from the current study, and future longitudinal studies are needed. Third, the reliance on self-reported data may pose a potential risk to the validity of the measurements. Finally, the R-squared values in the current study explained a small proportion of the variance, implying that other potential mediators between health literacy/social support and self-management may need to be considered in future studies.

CONCLUSION

The present study further explained the mechanism of health literacy/social support influences self-management. It reveals the importance of learned helplessness on self-management behavior change in MHD patients. This study's parallel mediator models contribute to developing and improving intervention strategies for MHD patients' health-related behaviors. It suggests that clinical caregivers should pay attention to preventing, identifying, and monitoring patients' learned helplessness when providing health education and medical support to MHD patients. Reducing

For peer review only - http://bmjopen.bmj.com/site/about/guidelines.xhtml

their helplessness and increasing internality enhances their motivation and persistence of self-management.

Acknowledgments

We express our heartfelt gratitude to all members who assisted in the data collection and processing and to every MHD patient who participated in this study.

Contributors

Chunyan Xie: Conceptualization, Data curation, Formal analysis, Writing – original draft. Li Li: Data curation, Formal analysis. Yamin Li: Writing - review & editing.

Funding

This research received grants from the National Natural Science Foundation of China (No.81974217), Major Scientific and Technological Projects in Hunan Province (No.2020SK208), and 2022 Central South University Graduate Student Independent Exploration and Innovation Program (No. 1053320213471).

Competing interests

All authors declare that they have no conflicts of interest.

Patient consent for publication

Obtained.

For peer review only - http://bmjopen.bmj.com/site/about/guidelines.xhtml

For peer review only

Ethics approval

The study was approved by the Ethics Committee of Xiangya Nursing School of Central South University on April 20, 2022 (No. E202255). Informed consent was obtained from all participants.

Provenance and peer review

Not commissioned; externally peer reviewed.

Data availability statement

The datasets analyzed during the current study are not publicly available due to them containing information that could compromise research participant consent but are available from the corresponding author on reasonable request.

Figure legends

Figure 1. Theoretical framework based on COM-B model. HL: Health Literacy; LH: Learned Helplessness; SS: Social Support; SM: Self-management.

Figure 2. Parallel multiple mediator models. A: Health Literacy Model (Model 1), B: Social Support Model (Model 2).

References

1. Bikbov B, Purcell C, Levey A, et al. GBD Chronic Kidney Disease Collaboration. Global, regional, and national burden of chronic kidney disease, 1990–2017: a systematic analysis for the Global Burden of Disease Study 2017. *Lancet* 2020;395(10225):709-33.

2. O'Hare AM. Dialysis adequacy reconsidered: The person comes first. *Seminars in dialysis*

For peer review only - http://bmjopen.bmj.com/site/about/guidelines.xhtml

2020;33(6):486-89. doi: 10.1111/sdi.12938 [published Online First: 2020/11/27]
3. Wang S-Y, Zang X-Y, Fu S-H, et al. Factors related to fatigue in Chinese patients with end-stage renal disease receiving maintenance hemodialysis: a multi-center cross-sectional study. *Renal failure* 2016;38(3):442-50.
4. He Y, Lu Y, Yang S, et al. Dietary Plant Protein and Mortality Among Patients Receiving Maintenance Hemodialysis: A Cohort Study. *American journal of kidney diseases : the official journal of the National Kidney Foundation* 2021;78(5):649-57.e1. doi: 10.1053/j.ajkd.2021.03.023 [published Online First: 2021/05/31]
5. Yorulmaz H, Bayraktar S, Ozdilli K. Posttraumatic growth in chronic kidney failure disease. *Proc Soc BehavSci* 2010;5:2313-19.
6. Brioni E, Delli Zotti G, Apuzzo L, et al. Self-efficacy and self-management in patients in hemodialysis: a narrative review of multidisciplinary theories. *G Ital Nefrol* 2021;38(3):2021-vol3. Italian.
7. Griva K, Nandakumar M, Jo-an HN, et al. Hemodialysis self-management intervention randomized trial (HED-SMART): a practical low-intensity intervention to improve adherence and clinical markers in patients receiving hemodialysis. *American Journal of Kidney Diseases* 2018;71(3):371-81.
8. Curtin RB, Sitter DC, Schatell D, et al. Self-management, knowledge, and functioning and well-being of patients on hemodialysis. *Nephrology Nursing Journal* 2004;31(4):378-86.
9. Tovazzi ME, Mazzoni V. Personal paths of fluid restriction in patients on hemodialysis. *Nephrology Nursing Journal* 2012;39(3):207-15.
10. Sun Q. Correlation between depressive conditions and self-management behaviors in maintenance hemodialysis patients (in Chinese). Liaoning University of Traditional Chinese Medicine, 2011.
11. Joboshi H, Oka M. Effectiveness of an educational intervention (the Encourage Autonomous Self-Enrichment Program) in patients with chronic kidney disease: a randomized controlled trial. *International journal of nursing studies* 2017;67:51-58.
12. Nutbeam D. The evolving concept of health literacy. *Social science medicine* 2008;67(12):2072-78.
13. Lopez-Vargas PA, Tong A, Howell M, et al. Educational interventions for patients with CKD: a systematic review. *American Journal of Kidney Diseases* 2016;68(3):353-70.
14. Devraj R, Borrego M, Vilay AM, et al. Relationship between health literacy and kidney function. *Nephrology* 2015;20(5):360-67.
15. Wu SFV, Hsieh NC, Lin LJ, et al. Prediction of self‐care behaviour on the basis of knowledge about chronic kidney disease using self‐efficacy as a mediator. *Journal of clinical nursing* 2016;25(17-18):2609-18.
16. Hu C, Lim P, Chang T. The effectiveness of case management on self-care and quality of life in chronic kidney disease patients. *The Journal of Health Science* 2012;14(1):13-28.
17. Tifferet S. Gender differences in social support on social network sites: A meta-analysis. *Cyberpsychology, behavior, social networking* 2020;23(4):199-209.
18. Cullen FT. Social support as an organizing concept for criminology: Presidential address to the Academy of Criminal Justice Sciences. *Justice Quarterly* 1994;11(4):527-59.
19. Cardol CK, Boslooper-Meulenbelt K, van Middendorp H, et al. Psychosocial barriers and facilitators for adherence to a healthy lifestyle among patients with chronic kidney disease: A focus group study. *BMC nephrology* 2022;23(1):1-16.
20. Neri L, Martini A, Andreucci VE, et al. Regimen complexity and prescription adherence in dialysis

For peer review only - http://bmjopen.bmj.com/site/about/guidelines.xhtml

patients. *American journal of nephrology* 2011;34(1):71-76.
21. Kauric-Klein Z. Predictors of nonadherence with blood pressure regimens in hemodialysis. *Patient preference adherence* 2013;7:973.
22. Song Y-y, Chen L, Wang W-x, et al. Social Support, Sense of Coherence, and Self-Management among Hemodialysis Patients. *Western Journal of Nursing Research* 2022;44(4):367-74.
23. Been‐Dahmen JM, Grijpma JW, Ista E, et al. Self‐management challenges and support needs among kidney transplant recipients: A qualitative study. *Journal of advanced nursing* 2018;74(10):2393-405.
24. Camacho EM, Verstappen SM, Chipping J, et al. Learned helplessness predicts functional disability, pain and fatigue in patients with recent-onset inflammatory polyarthritis. *Rheumatology (Oxford, England)* 2013;52(7):1233-8. doi: 10.1093/rheumatology/kes434 [published Online First: 2013/02/21]
25. Crielaard L, Nicolaou M, Sawyer A, et al. Understanding the impact of exposure to adverse socioeconomic conditions on chronic stress from a complexity science perspective. *BMC medicine* 2021;19(1):242. doi: 10.1186/s12916-021-02106-1 [published Online First: 2021/10/13]
26. Peterson C, Seligman ME, Vaillant GE. Pessimistic explanatory style is a risk factor for physical illness: a thirty-five-year longitudinal study. *Journal of personality and social psychology* 1988;55(1):23-7. doi: 10.1037//0022-3514.55.1.23 [published Online First: 1988/07/01]
27. Yu-Ying C, Li-Ya T. Applying Swanson's Caring Theory to a Patient With End Renal Stage Disease Experiencing Hopelessness. *Journal of Nursing* 2021;68(2):99-106. doi: 10.6224/JN.202104_68(2).13
28. Kurita N, Wakita T, Fujimoto S, et al. Hopelessness and Depression Predict Sarcopenia in Advanced CKD and Dialysis: A Multicenter Cohort Study. *The journal of nutrition, health & aging* 2021;25(5):593-99. doi: 10.1007/s12603-020-1556-4 [published Online First: 2021/05/06]
29. Khalil AA, Abed MA. Perceived social support is a partial mediator of the relationship between depressive symptoms and quality of life in patients receiving hemodialysis. *Archives of Psychiatric Nursing* 2014;28(2):114-18.
30. Bayuo J, Bristowe K, Harding R, et al. "Managing uncertainty": Experiences of family members of burn patients from injury occurrence to the end-of-life period. *Burns : journal of the International Society for Burn Injuries* 2021;47(7):1647-55. doi: 10.1016/j.burns.2021.02.009 [published Online First: 2021/04/10]
31. Smallheer BA, Vollman M, Dietrich MS. Learned helplessness and depressive symptoms following myocardial infarction. *Clin Nurs Res* 2018;27(5):597-616.
32. Kail BL, Carr DC. Successful aging in the context of the disablement process: Working and volunteering as moderators on the association between chronic conditions and subsequent functional limitations. *The Journals of Gerontology: Series B* 2017;72(2):340-50.
33. Michie S, Van Stralen MM, West R. The behaviour change wheel: a new method for characterising and designing behaviour change interventions. *Implementation science* 2011;6(1):1-12.
34. Murtagh E, Barnes A, McMullen J, et al. Mothers and teenage daughters walking to health: using the behaviour change wheel to develop an intervention to improve adolescent girls' physical activity. *Public health* 2018;158:37-46.
35. Li H. Research on the Condition and Influencing Factors ofHemodialysis Patients' Self Management. Peking Union Medical College, 2011.

For peer review only - http://bmjopen.bmj.com/site/about/guidelines.xhtml

36. Xiao SY. The theoretical basis and research application of the Social Support Rating Scale (in Chinese). *Journal of Clinical Psychiatry* 1994;99(04):98–100.
37. Song YJ. Construction and testing of a self-management scale for hemodialysis patients (in Chinese). Kaohsiung Medical University, 2009.
38. Li H, Cao YD, Jiang YF, et al. Introduction and reliability testing of a self-management scale for hemodialysis patients (in Chinese). *Chin J Nurs* 2015;50(11):1392-95.
39. Nicassio PM, Wallston KA, Callahan LF, et al. The measurement of helplessness in rheumatoid arthritis. The development of the arthritis helplessness index. *J Rheumatol* 1985;12(3):462-67.
40. Pan W, Gao B, Long Y, et al. Effect of Caregivers' Parenting Styles on the Emotional and Behavioral Problems of Left-Behind Children: The Parallel Mediating Role of Self-Control. 2021;18(23):12714.
41. Mills S, Azizoddin D, Gholizadeh S, et al. The mediational role of helplessness in psychological outcomes in systemic lupus erythematosus. *Lupus* 2018;27(7): 1185-89.
42. Van Der Werf SP, Evers A, Jongen PJH, et al. The role of helplessness as mediator between neurological disability, emotional instability, experienced fatigue and depression in patients with multiple sclerosis. *Multiple Sclerosis Journal* 2003;9(1):89-94.
43. Kuttner MJ, Delamater AM, Santiago JV. Learned helplessness in diabetic youths. *Journal of Pediatric Psychology* 1990;15(5):581-94.
44. DeVellis R, Callahan L. A brief measure of helplessness in rheumatic disease: the helplessness subscale of the Rheumatology Attitudes Index. *The Journal of rheumatology* 1993;20(5):866-69.
45. Rotter JB. Generalized expectancies for internal versus external control of reinforcement. *Psychol Monogr Gen Appl* 2010;80:1–28.
46. Seligman M, Weiss J, Weinraub M, et al. Coping behavior: learned helplessness, physiological change and learned inactivity. *Behaviour research therapy* 1980;18(5):459-512. doi: 10.1016/0005-7967(80)90011-x
47. Neuvonen E, Lehtisalo J, Solomon A, et al. Psychosocial determinants for adherence to a healthy lifestyle and intervention participation in the FINGER trial: an exploratory analysis of a randomised clinical trial. *Aging clinical and experimental research* 2022;34(8):1793-805. doi: 10.1007/s40520-022-02088-x [published Online First: 2022/02/20]
48. Sheridan N, Kenealy T, Salmon E, et al. Helplessness, self blame and faith may impact on self management in COPD: a qualitative study. *Primary Care Respiratory Journal* 2011;20(3):307-14.
49. Omachi TA, Katz PP, Yelin EH, et al. The COPD Helplessness Index: a new tool to measure factors affecting patient self-management. *Chest* 2010;137(4):823-30. doi: 10.1378/chest.09-0764 [published Online First: 2009/10/20]
50. Wang LM, Chiou CP. Effectiveness of interactive multimedia CD on self-care and powerlessness in hemodialysis patients. *Journal of Nursing Research* 2011;19(2):102-11.
51. Fan JL, Kong Y, Shi SH, et al. Correlation Study of Health Locus of Control and Self-management Behaviors in Patients Underwent Hemodialysis (in Chinese). *Nursing Journal of Chinese People's Liberation Army* 2016;33(9):23-27. doi: 10.3969/j.issn.1008-9993.2016.09.005
52. Lael-Monfared E, Tehrani H, Teiho Z, et al. The study of eye care behaviors in patients with type 2 diabetes. *Journal of Diabetes Metabolic Disorders* 2020;19(1):257-63.

For peer review only - http://bmjopen.bmj.com/site/about/guidelines.xhtml

53. Chen CC, Li HP, Liu HQ, et al. A study on the relationship among self - management behavior, family function, locus of control among maintenance hemodialysis patient (in Chinese). *Chinese Journal of health education* 2020;36(11):1053.

54. Kiajamali M, Hosseini M, Estebsari F, et al. Correlation between social support, self-efficacy and health-promoting behavior in hemodialysis patients hospitalized in Karaj in 2015. *Electronic Physician* 2017;9(7):4820.

55. Abramson LY, Seligman ME, Teasdale JD. Learned helplessness in humans: critique and reformulation. *Journal of abnormal psychology* 1978;87(1):49.

For peer review only

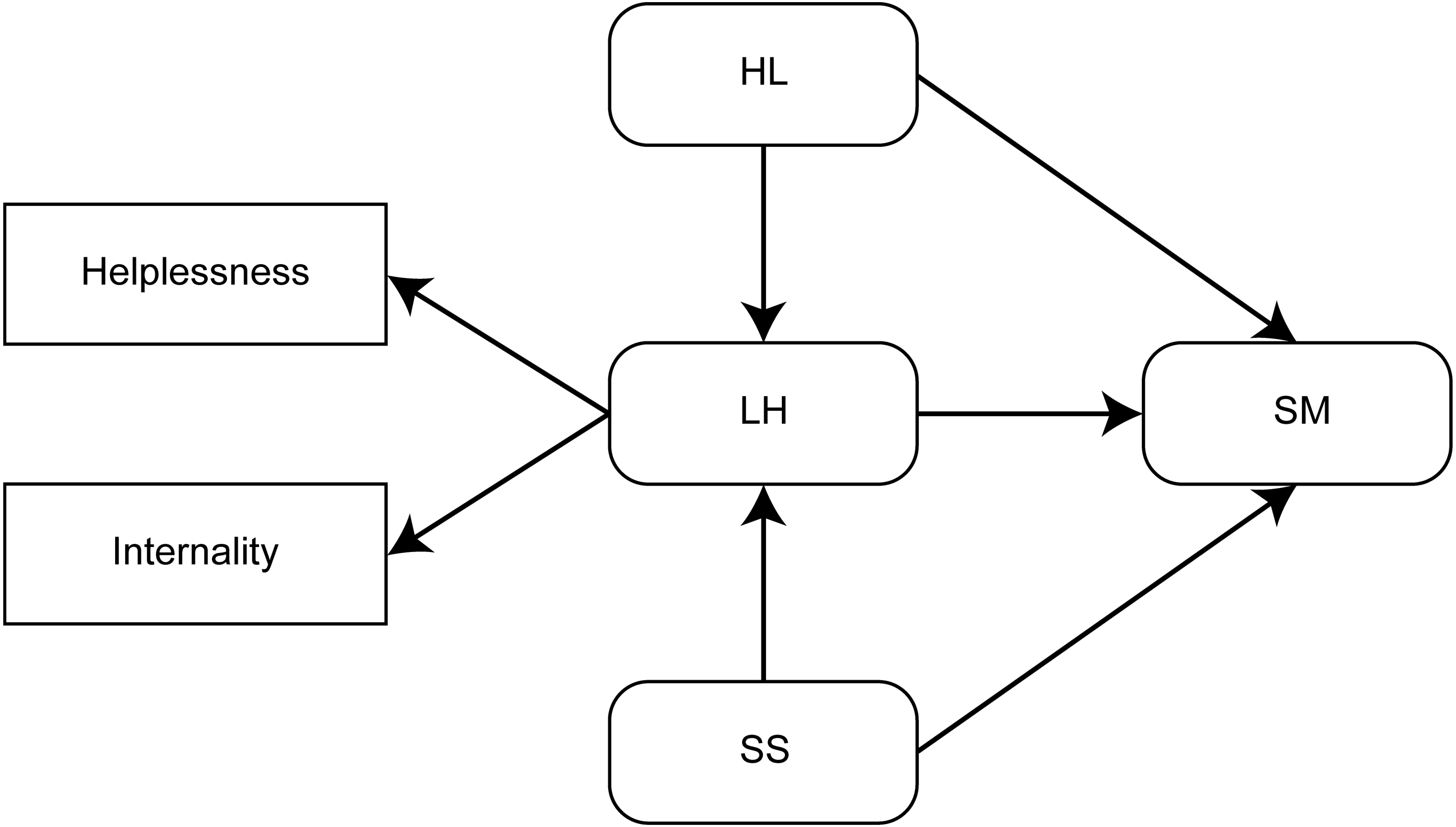
HL
Helplessness
LH
SM
Internality
SS

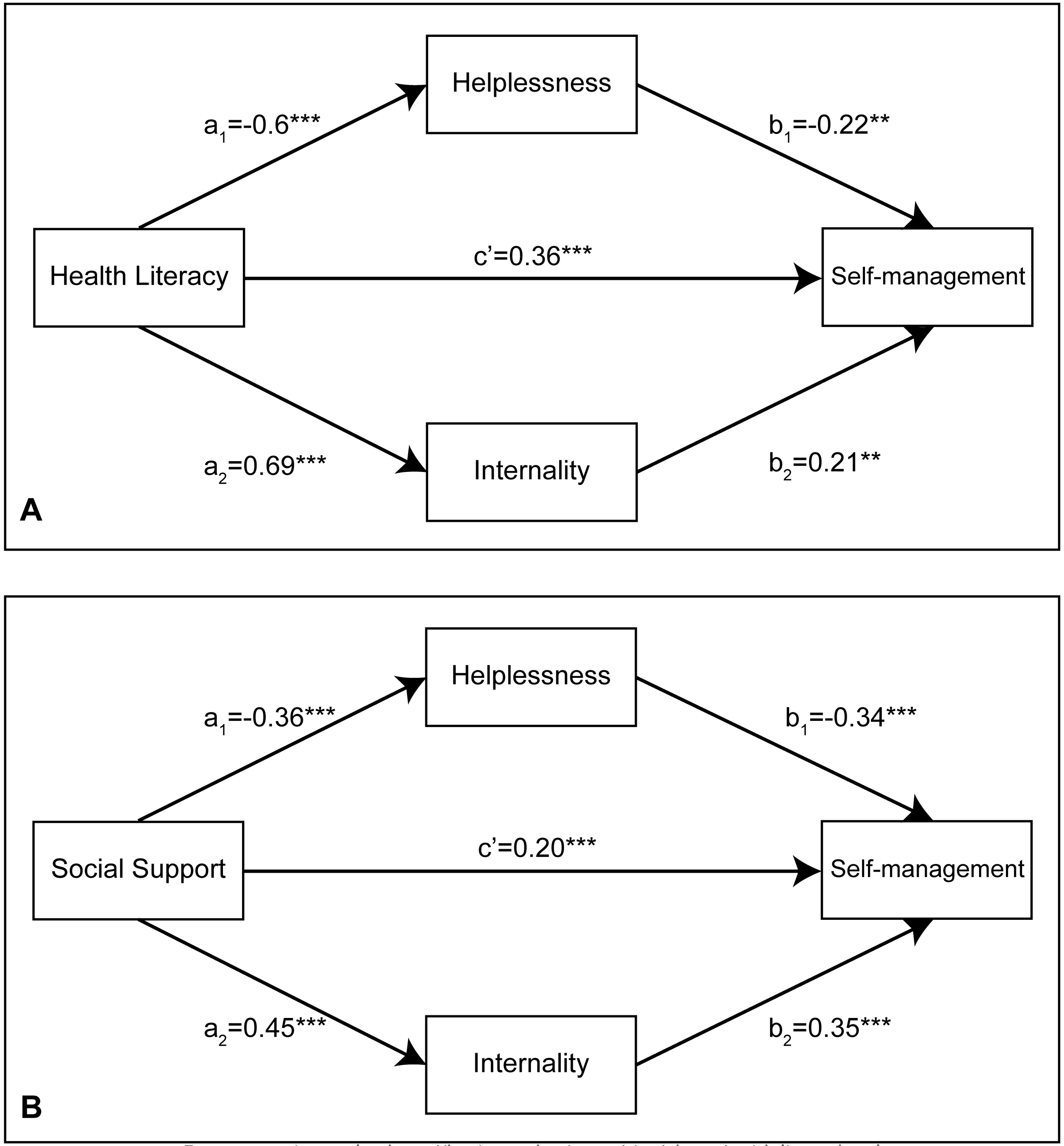
Helplessness
Health Literacy
c’=0.36***
Self-management
Internality
a1=-0.6***
b1=-0.22**
a2=0.69***
b2=0.21**
A
Helplessness
Social Support
c’=0.20***
Self-management
Internality
a1=-0.36***
b1=-0.34***
a2=0.45***
b2=0.35***
B

Reporting checklist for cross sectional study.

Based on the STROBE cross sectional guidelines.

Instructions to authors

Complete this checklist by entering the page numbers from your manuscript where readers will find each of the items listed below.

Your article may not currently address all the items on the checklist. Please modify your text to include the missing information. If you are certain that an item does not apply, please write "n/a" and provide a short explanation.

Upload your completed checklist as an extra file when you submit to a journal.

In your methods section, say that you used the STROBE cross sectionalreporting guidelines, and cite them as:

von Elm E, Altman DG, Egger M, Pocock SJ, Gotzsche PC, Vandenbroucke JP. The Strengthening the Reporting of Observational Studies in Epidemiology (STROBE) Statement: guidelines for reporting observational studies.

		Reporting Item	Page Number
**Title and abstract**			
Title	#1a	Indicate the study's design with a commonly used term in the title or the abstract	1
Abstract	#1b	Provide in the abstract an informative and balanced summary of what was done and what was found	1
**Introduction**			
Background / rationale	#2	Explain the scientific background and rationale for the investigation being reported	2-7
Objectives	#3	State specific objectives, including any prespecified hypotheses	7
**Methods**			

For peer review only

Study design	#4	Present key elements of study design early in the paper	8
Setting	#5	Describe the setting, locations, and relevant dates, including periods of recruitment, exposure, follow-up, and data collection	8
Eligibility criteria	#6a	Give the eligibility criteria, and the sources and methods of selection of participants.	8
	#7	Clearly define all outcomes, exposures, predictors, potential confounders, and effect modifiers. Give diagnostic criteria, if applicable	N/A
Data sources / measurement	#8	For each variable of interest give sources of data and details of methods of assessment (measurement). Describe comparability of assessment methods if there is more than one group. Give information separately for for exposed and unexposed groups if applicable.	8-10
Bias	#9	Describe any efforts to address potential sources of bias	N/A
Study size	#10	Explain how the study size was arrived at	N/A
Quantitative variables	#11	Explain how quantitative variables were handled in the analyses. If applicable, describe which groupings were chosen, and why	10
Statistical methods	#12a	Describe all statistical methods, including those used to control for confounding	10
Statistical methods	#12b	Describe any methods used to examine subgroups and interactions	10
Statistical methods	#12c	Explain how missing data were addressed	N/A
Statistical methods	#12d	If applicable, describe analytical methods taking account of sampling strategy	N/A
Statistical methods	#12e	Describe any sensitivity analyses	N/A
**Results**			
Participants	#13a	Report numbers of individuals at each stage of study—eg numbers potentially eligible, examined for eligibility, confirmed	N/A

For peer review only

		eligible, included in the study, completing follow-up, and analysed. Give information separately for for exposed and unexposed groups if applicable.	
Participants	#13b	Give reasons for non-participation at each stage	N/A
Participants	#13c	Consider use of a flow diagram	N/A
Descriptive data	#14a	Give characteristics of study participants (eg demographic, clinical, social) and information on exposures and potential confounders. Give information separately for exposed and unexposed groups if applicable.	11
Descriptive data	#14b	Indicate number of participants with missing data for each variable of interest	N/A
Outcome data	#15	Report numbers of outcome events or summary measures. Give information separately for exposed and unexposed groups if applicable.	11-12
Main results	#16a	Give unadjusted estimates and, if applicable, confounder-adjusted estimates and their precision (eg, 95% confidence interval). Make clear which confounders were adjusted for and why they were included	N/A
Main results	#16b	Report category boundaries when continuous variables were categorized	N/A
Main results	#16c	If relevant, consider translating estimates of relative risk into absolute risk for a meaningful time period	N/A
Other analyses	#17	Report other analyses done—e.g., analyses of subgroups and interactions, and sensitivity analyses	11-12
**Discussion**			
Key results	#18	Summarise key results with reference to study objectives	13-17
Limitations	#19	Discuss limitations of the study, taking into account sources of potential bias or imprecision. Discuss both direction and magnitude of any potential bias.	17-18
Interpretation	#20	Give a cautious overall interpretation considering objectives, limitations, multiplicity of analyses, results from similar studies, and other relevant evidence.	13-17

For peer review only

Generalisability	#21	Discuss the generalisability (external validity) of the study results	18
**Other Information**			
Funding	#22	Give the source of funding and the role of the funders for the present study and, if applicable, for the original study on which the present article is based	19

The STROBE checklist is distributed under the terms of the Creative Commons Attribution License CC-BY. This checklist was completed on 23. September 2022 using https://www.goodreports.org/, a tool made by the EQUATOR Network in collaboration with Penelope.ai

For peer review only

BMJ Open

The mediating role of learned helplessness's components in the association between health literacy/social support and self-management among maintenance hemodialysis patients in Changsha, China: a cross-sectional study

Journal:	*BMJ Open*
Manuscript ID	bmjopen-2022-068601.R1
Article Type:	Original research
Date Submitted by the Author:	14-Mar-2023
Complete List of Authors:	Xie, Chunyan; Central South University, Xiangya Nursing School<br>Li, Li; The Second Xiangya Hospital of Central South University, Clinical Nursing Teaching and Research Section<br>Li, Ya-Min; The Second Xiangya Hospital of Central South University, Clinical Nursing Teaching and Research Section
**Primary Subject Heading**:	Mental health
Secondary Subject Heading:	Nursing, Mental health
Keywords:	MENTAL HEALTH, Dialysis < NEPHROLOGY, Change management < HEALTH SERVICES ADMINISTRATION & MANAGEMENT

SCHOLARONE™
Manuscripts

I, the Submitting Author has the right to grant and does grant on behalf of all authors of the Work (as defined in the below author licence), an exclusive licence and/or a non-exclusive licence for contributions from authors who are: i) UK Crown employees; ii) where BMJ has agreed a CC-BY licence shall apply, and/or iii) in accordance with the terms applicable for US Federal Government officers or employees acting as part of their official duties; on a worldwide, perpetual, irrevocable, royalty-free basis to BMJ Publishing Group Ltd ("BMJ") its licensees and where the relevant Journal is co-owned by BMJ to the co-owners of the Journal, to publish the Work in this journal and any other BMJ products and to exploit all rights, as set out in our licence.

The Submitting Author accepts and understands that any supply made under these terms is made by BMJ to the Submitting Author unless you are acting as an employee on behalf of your employer or a postgraduate student of an affiliated institution which is paying any applicable article publishing charge ("APC") for Open Access articles. Where the Submitting Author wishes to make the Work available on an Open Access basis (and intends to pay the relevant APC), the terms of reuse of such Open Access shall be governed by a Creative Commons licence – details of these licences and which Creative Commons licence will apply to this Work are set out in our licence referred to above.

Other than as permitted in any relevant BMJ Author's Self Archiving Policies, I confirm this Work has not been accepted for publication elsewhere, is not being considered for publication elsewhere and does not duplicate material already published. I confirm all authors consent to publication of this Work and authorise the granting of this licence.

For peer review only

The mediating role of learned helplessness's components in the association between health literacy/social support and self-management among maintenance hemodialysis patients in Changsha, China: a cross-sectional study

Chunyan Xie[1,2], Li Li[2,3], Yamin Li[1]

[1] Clinical Nursing Teaching and Research Section, The Second Xiangya Hospital, Central South University, Changsha, Hunan, 410011, People's Republic of China;

[2] XiangYa Nursing School, Central South University, Changsha, Hunan, 410013, People's Republic of China;

[3] The First Affiliated Hospital of Xinjiang Medical University, Urumqi, Xinjiang, 830011, People's Republic of China

Correspondence: Yamin Li, The Second Xiangya Hospital, Central South University, Changsha, Hunan, 410011, People's Republic of China, Email aminny@csu.edu.cn

Abstract

Objectives: To explore the multiple mediating roles of the learned helplessness's core system in the relationship between health literacy/social support and self-management.

Design: Cross-sectional survey design.

Setting: Changsha, China.

Participants: 239 Chinese maintenance hemodialysis (MHD) patients.

For peer review only - http://bmjopen.bmj.com/site/about/guidelines.xhtml

Methods: Two multiple mediator models were constructed based on the COM-B model. A total of 239 Chinese MHD patients participated in a cross-sectional study, which included surveys on the Learned Helplessness Scale for MHD patients, Dialysis Knowledge Questionnaire, Social Support Scale, and Self-Management Scale for Hemodialysis. The PROCESS macro in SPSS was used for mediated effects analysis.

Results: Helplessness and internality partially mediated the relationship between health literacy/social support and self-management [(β = -0.212, $P < 0.01$; β = 0.240, $P < 0.01$)/(β = -0.331, $P < 0.001$; β = 0.376, $P < 0.001$)]. The mediation effect size was 0.7802 (95% CI [0.373, 1.218]) in the health literacy model, accounting for 45.29% of the total effect, and 0.2857 (95% CI [0.207, 0.377]) in the social support model, accounting for 57.88% of the total effect. The differences in effect sizes for helplessness and internality in the two models were -0.080 (95% CI [-0.374, 0.216]) and -0.041 (95% CI [-0.127, 0.043]), respectively.

Conclusion: Health literacy/social support directly affects MHD patients' self-management and indirectly affects it by changing learned helplessness, such as increasing internality while reducing helplessness.

Keywords: learned helplessness, self-management, health literacy, social support, multiple mediations

Strengths and limitations of this study:

For peer review only

For peer review only - http://bmjopen.bmj.com/site/about/guidelines.xhtml

1. The PROCESS macro in SPSS was used to verify the potential mediating effects of learned helplessness's components on the relationship between health literacy/social support and self-management in Chinese maintenance hemodialysis patients.

2. A culturally adapted and validated Chinese version of the Learned Helplessness Scale for Maintenance Hemodialysis Patients was used.

3. Based on the ethnic diversity of China, participants could be recruited from a more diverse range of cultures and backgrounds.

4. Causality cannot be inferred from the current study, and future longitudinal studies are needed.

5. The self-reported data may pose a potential risk to the validity of the measurements.

BACKGROUND

As of 2017, there were approximately 700 million patients with chronic kidney disease (CKD) worldwide, with nearly one-third of them concentrated in China and India (132 million and 115 million, respectively) [1]. CKD is divided into five stages based on the glomerular filtration rate. CKD's fifth stage is called end-stage kidney disease (ESKD), when the patient's renal function has irreversibly declined or wholly lost. The main treatments to maintain ESKD patients' lives are hemodialysis, peritoneal dialysis, and renal transplantation. Maintenance hemodialysis (MHD) is the most widely used alternative treatment in clinical practice[2 3]. MHD is defined as

For peer review only - http://bmjopen.bmj.com/site/about/guidelines.xhtml

regular hemodialysis patients receiving for more than three months with a frequency of 2-3 times per week[4]. In China, most ESKD patients are treated with MHD[5].

Although MHD can prolong their lives, it cannot completely replace renal function. Patients must also slow their disease progression through self-management, such as long-term medications, restricted diet, fluid intake, regular exercise, and arteriovenous fistula care[6 7]. MHD patients' self-management means they are proactive in controlling their diet, performing self-care, and managing the disease with their caregivers to prevent complications and achieve desired life goals[8]. Curtin et al.[8] showed that self-management behaviours positively impacted MHD patients' overall health status. However, a large number of studies point to the generally unsatisfactory self-management status of MHD patients. For example, less than 40% of them can strictly follow the doctor's advice to control their diet and water[9]. The overall level of Chinese MHD patients' self-management is low, with a score of 45.21 ± 13.44 (total scale score of 80)[10]. Effective implementation of self-management requires a combination of multiple factors, such as the patient's sex, age, dialysis duration, and frequency. However, these relevant factors are not easily changed. Therefore, it has become a priority for healthcare staff to investigate the controllable factors influencing MHD patients' self-management to improve their health status and quality of life.

Health literacy/social support and self-management

For peer review only - http://bmjopen.bmj.com/site/about/guidelines.xhtml

A growing body of research has focused on how to improve MHD patients' self-management behaviours. Health literacy is an important influencing factor for improving self-management behaviours[11], which reflects the degree of patients' awareness of the disease and determines their motivation and ability to acquire, understand, and use knowledge to improve their health[12]. Notably, nursing education aims to improve patients' perceptions of disease and overall health literacy to induce changes in their behaviors[13]. When patients are more aware of their disease and health, they become aware of the importance of self-management and are more likely to adopt healthy behaviors, as confirmed by more studies. For example, Devraj et al.[14] showed that health literacy in CKD patients was positively associated with self-management, and a randomized controlled trial in Japan confirmed similar results[11]. Wu et al.[15] found that health literacy could influence CKD patients' self-management through the mediating role of self-efficacy, which is consistent with Hu et al.[16].

Second, social support is a factor that significantly impacts the treatment of most patients with chronic diseases[17]. It is a multidimensional concept that refers to the spiritual and material support given to individuals by various parties, such as family, relatives, friends, and social organizations[18]. Adequate social support can reduce MHD patients' financial or psychological burdens and enable them to manage life better[19]. Several studies[20 21] have shown that the lack of family support in MHD patients is followed by poor medication adherence. Song et al.[22] used a structural equation modelling approach to confirm that social support directly affects MHD

For peer review only - http://bmjopen.bmj.com/site/about/guidelines.xhtml

patients' self-management and, together with the sense of consistency, explained 69% of the variance in self-management behavior. Been-Dahmen et al.[23] noted through qualitative interviews that family, friends, professionals, and colleagues also provide essential emotional support to kidney transplant patients. Thus, social support is crucial for MHD patients to achieve their health goals.

Although previous studies have confirmed the relationship between health literacy, social support, and self-management, most studies have overlooked possible potential pathways for this relationship. Therefore, the study was dedicated to exploring the multiple potential possibilities between health literacy/social support and self-management.

Learned helplessness

Seligman first observed learned helplessness (LH) in 1967[24]. LH is described as a psychological state of powerlessness or self-abandonment when the individual perceives a disconnection between behavior and outcome, manifested by cognitive deficits, decreased motivation, and emotional maladjustment[25 26]. Learned helplessness is often used to explain the phenomenon of diminished or absent motivation in individuals and extends to health-related problems[27]. For example, helpless patients adhere significantly less to regular treatment and seem to have more difficulty carrying out physicians' recommendations for diet, water, and exercise[28 29]. Learned helplessness was divided into two core components. Helplessness is the individual's perception that their behavior cannot impact the outcome[30]. Internality

For peer review only - http://bmjopen.bmj.com/site/about/guidelines.xhtml

refers to the individual's perception that they believe that they are the most dominant force dominating their life[31].

It has been suggested that learned helplessness is associated with low social support and health literacy. First, adequate social support gives patients more tangible resources for life and intangible resources for spirituality, which can buffer the multiple negative emotions associated with illness[32]. Those with high social support have access to disease-related knowledge through multiple channels to reduce helplessness when coping with the disease. Notably, family support always plays a vital role in reducing patients' learned helplessness[33]. Smallheer et al.[34] suggested that emotional support from family and friends, access to disease-related knowledge, and trust and support with fellow patients and healthcare staff can help prevent patients' helplessness. In addition, it has been suggested that social support and health literacy can help maintain physical and mental harmony and influence helplessness by integrating them[35].

Theoretical basis

The competence, opportunity, and motivation-behavior (COM-B) model is a theoretical model related to individual behavior change proposed by Michie[36]. The COM-B model explains that competence, opportunity, and motivation can influence an individual's behavior, whereas competence and opportunity can influence the individual's behavior indirectly through motivation[37]. According to this model, although people with MHD are willing to engage in self-management, they give little

For peer review only - http://bmjopen.bmj.com/site/about/guidelines.xhtml

action, which may be closely related to their ability, opportunity, and motivation. Capacity refers to individuals' knowledge and skills to engage in behavior change[37]. Opportunity refers to the external factors that promote behavior change[37]. Motivation refers to the beliefs that guide an individual's behavior change[37]. In this study, the good self-management behaviours exhibited by the patients first required a certain knowledge and skills of kidney disease care, i.e., dialysis-related health literacy. At the opportunity level, social support from family members, friends, and healthcare providers gives emotional and material security to patients. However, chronic learned helplessness can undermine patients' motivation to engage in self-management and affect their proactive health behaviors.

After a literature review and theoretical analysis, this study finally developed a mediating model with two core systems of learned helplessness as motivational factors, using social support as an opportunity factor, health literacy as a competence factor, and self-management as a dependent variable. (As shown in Figure 1)

The current study

There may be a relationship between health literacy/social support, learned helplessness, and self-management behaviours. However, the underlying mechanisms between these relationships remain to be further identified. In this study, we developed two multiple mediator models based on the COM-B model to help us understand the role of learned helplessness in forming and changing health behaviors. To test this, we made the following hypotheses.

For peer review only - http://bmjopen.bmj.com/site/about/guidelines.xhtml

Hypothesis 1 (H1): Health literacy/social support can influence two dimensions of learned helplessness (helplessness, internality).

Hypothesis 2 (H2): Two dimensions of learned helplessness (helplessness, internality) predict self-management.

Hypothesis 3 (H3): Two dimensions of learned helplessness (helplessness, internality) parallelly mediate the relationship between health literacy/social support and self-management.

METHODS

Study design and participants

This is a cross-sectional study to investigate the association between health literacy/social support and self-management through the mediating role of the component of lean helplessness. This study followed the Strengthening the Reporting of Observational Studies in Epidemiology Checklist for cross-sectional studies (STROBE 2007, Supplementary File 1) guidelines to ensure the quality and specification of study reporting.

We used convenience sampling to select patients from a comprehensive Grade 3A hospital in Changsha, Hunan Province, China, who met the following criteria: (i) being treated with hemodialysis for ≥ 3 months, (ii) being informed and agreeing to participate in this study, and (iii) being conscious, being literate and having communication abilities. We excluded patients with mental illness, cognitive dysfunction, or serious complications. The purpose and significance of this study

For peer review only - http://bmjopen.bmj.com/site/about/guidelines.xhtml

were clarified to the participants before the survey to obtain informed consent. The questionnaires were administered 1 hour after the start or 1 hour before the end of dialysis because the patients' blood circulation was more stable. Each participant took approximately 10-15 minutes to complete the questionnaire.

Measures

Health Literacy

We used the Dialysis Knowledge Questionnaire developed by Curtin[8] to measure MHD patients' health literacy. The questionnaire was divided into five dimensions after cross-cultural adaptation by Li[38]. It includes anemia, diet and medication, kidney function, dialysis, and rehabilitation, with 24 items. The patients were asked to judge whether the items' descriptions were true, scoring 1 for a correct answer and 0 for an incorrect/unknown answer. The total score was 24. The higher the score, the higher the patient's knowledge of dialysis. The questionnaire's content validity index (CVI) was 0.94, and the Cronbach's alpha coefficient was 0.701.

Social support

The Social Support Rating Scale (SSRS) developed by Xiao[39] was selected for this study. The scale consists of ten items involving three dimensions: objective support, subjective support, and support utilization. Items 1-4 and 8-10 were scored on a 4-point Likert scale. Item 5 has five subitems, each of which has four options from "none" to "fully support," scoring 1-4 points. The scores of items 6 and 7 are

For peer review only - http://bmjopen.bmj.com/site/about/guidelines.xhtml

calculated according to the number of support sources. The total score ranges from 12 to 66, with higher scores indicating higher levels of social support. The Cronbach's alpha coefficient for the total scale was 0.896. This instrument has been used in Chinese renal dialysis patients[22 40].

Self-management

The Self-Management Scale for Hemodialysis (SMSH), developed by Song[41] in 2009, was used to measure MHD patients' self-management behaviors in this study. It has 20 items and is divided into four dimensions: problem solving, performing self-care, partnership, and emotional processing. The scale was scored on a 4-point Likert scale, with scores 1-4 representing the frequency of the behavior, i.e., never, occasionally, often, and always, respectively. The total score was 20-80. The higher the score, the better the patient's self-management behaviour. It was revised by Chinese Li[42], and its Cronbach's alpha coefficient was 0.813.

Learned helplessness

There is no special scale for MHD patients' LH. Considering this limitation, we formed a tool called LHS-MHD based on Nicassio's AHI[43] scale. Under his authorization, we conducted cross-cultural adaptation, psychological measurement, and evaluation. The LHS-MHD has 11 items and was classified into two dimensions (helplessness/internality) based on the results of exploratory factor analysis (EFA) and confirmatory factor analysis (CFA), in line with the Learned Helplessness Theory. It

For peer review only - http://bmjopen.bmj.com/site/about/guidelines.xhtml

used a five-point Likert scale with response options ranging from 1 = strongly disagree to 5 = strongly agree. Six items in internality are reverse scored. The higher the total score, the more severe the learned helplessness. In our previous study, the scale showed good internal consistency [(Cronbach's α of 0.759), retest reliability (n=30, intragroup correlation=0.772), and split-half reliability [0.774][44].

Statistical analysis

We checked the data integrity and processed missing values before analysis. Expection-maximization or regression algorithms were used to produce estimates instead of missing values. Questionnaires with missing values exceeding 10% of the total items were considered invalid and given for exclusion. IBM SPSS version 25 was used to complete the descriptive statistics and correlation analysis of all variables. If the data conformed to a normal distribution, the Pearson correlation coefficient was used to express the correlation between the two variables. The PROCESS macro in SPSS, developed by Hayes (2013), was used for multiple mediation analyses. We adopted a bootstrap method (sampling was repeated 5000 times) to construct a 95% confidence interval for significance testing of mediating effects.

This study used three regression models to examine the parallel mediators' role of helplessness and internality in the relationship between health literacy/social support and self-management. Model 1, with health literacy/social support as the independent variable and helplessness as the dependent variable. In model 2, health literacy/social support remained the independent variable, while internality was the

For peer review only

dependent variable. Model 3 used health literacy/social support as the independent variable, helplessness and internality as mediating variables, and self-management as the dependent variable. Control variables such as age and gender were introduced in the model as covariates.

Patient and public involvement

Patients and/or the public were not involved in the design, conduct, reporting, or dissemination plans of this research.

RESULTS

Demographic characteristics

The study population consisted of 239 Chinese MHD patients aged 21-86 with a mean age of 55.61 and a standard deviation (SD) = 13.31. Among them, 59.8% were male. The characteristics of the participants are shown in Table 1.

Table 1 Descriptive statistics of sociodemographic characteristics (n=239).

Variable		n	%
Gender			
	Male	143	59.8
	Female	96	40.2
Age (years)			
	18-44	46	19.2
	45-59	100	41.8
	≥60	93	38.9
Education level			
	Elementary school	26	10.9
	Secondary school	126	52.7
	University and above	87	36.4
Employed status			
	On duty	10	4.2

For peer review only - http://bmjopen.bmj.com/site/about/guidelines.xhtml

	Part-time job	37	15.5
	Off duty	192	80.3
Protopathy			
	Hypertension	52	21.8
	Diabetes	48	20.1
	Chronic nephritis	105	43.9
	Polycystic kidney	20	8.4
	Others	14	5.9
Duration Of the MHD（years）			
	＜1	54	22.6
	1-3	42	17.6
	3-7	72	30.1
	≥ 7	71	29.7

Preliminary analysis

The main variables were normally distributed, so Pearson correlation analysis was performed to test the relationships among the measured variables. The correlations between the variables are listed in Table 2. The results of the correlation analyses were consistent with the hypotheses. All of them were statistically significant at the *P*<0.05 level (two-tailed). First, social support, health literacy, and self-management were all negatively correlated with helplessness (r = -0.361, -0.599, and -0.463). Second, internality was positively associated with social support, health literacy, and self-management, with correlation coefficients of 0.445, 0.691, and 0.489, respectively. Third, social support and health literacy positively predicted self-management (r = 0.48, 0.636).

Table 2 Descriptive statistics and Pearson's correlation coefficients for all variables of interest (n=239).

Variable	M	SD	1	2	3	4	5

For peer review only

For peer review only - http://bmjopen.bmj.com/site/about/guidelines.xhtml

1.Helplessness	16.858	4.70	1				
2.Internality	17.351	6.411	-.140*	1			
3.Social Support	44.628	10.094	-.361**	.445**	1		
4.Self-Management	53.197	10.537	-.463**	.489**	.480**	1	
5.Health Literacy	16.889	3.921	-.599**	.691**	.443**	.636**	1

Note: **p*<0.05，** *p* < 0.01. M=mean, SD=standard deviation.

For peer review only

Parallel multiple mediator models

This study used three regression models to examine the parallel mediators' role of helplessness and internality in the relationship between health literacy/social support and self-management (Table 3 and Figure 2). The results of Model 1 showed that health literacy/social support was negatively associated with helplessness (β = -0.616, *P* < 0.001; β = -0.354, *P* < 0.001). In model 2, health literacy/social support was positively associated with internality (β = 0.666, *P* < 0.001; β = 0.416, *P* < 0.001). In Model 3, we found that in the health literacy model, the effects of both helplessness and internality on self-management were significant (β = -0.212, *P* < 0.01; β = 0.240, *P* < 0.01), and health literacy remained a significant positive predictor of self-management (β = 0.351, *P* < 0.001). Similarly, in the social support model, social support, helplessness, and internality all had significant effects on self-management (β = 0.199, *P* < 0.001; β = -0.331, *P* < 0.001; β = 0.376, *P* < 0.001). Therefore, we deduced that helplessness and internality partially mediate the relationship between health literacy/social support and self-management.

Table 3 Results of the multiple mediation analysis.

For peer review only - http://bmjopen.bmj.com/site/about/guidelines.xhtml

Regression Model		Fitting Indices		Regression Coefficient	
Outcome Variable	Predictor Variable	R^2	F	β	t
Helplessness	Social Support	0.402	6.357	-0.354	-5.808***
Internality	Social Support	0.523	12.422	0.416	7.337***
	Social Support			0.199	3.411***
Self-management	Helplessness	0.668	20.526	-0.331	-6.172***
	Internality			0.376	6.522***
Helplessness	Health Literacy	0.629	21.630	-0.616	-11.682***
Internality	Health Literacy	0.722	35.981	0.666	14.197***
	Health Literacy			0.351	3.635***
Self-management	Helplessness	0.671	20.828	-0.212	-2.998**
	Internality			0.240	3.028**

*Note: *p < 0.05, **p < 0.01, ***p < 0.001.*

To confirm the significance of the model's total, direct, and indirect effects, we used a bootstrap analysis with 5000 repeated sampling tests, and the confidence interval was set at 95%. The results showed significant parallel mediating effects of helplessness and internality on the relationship between health literacy/social support and self-management. The indirect effect size of health literacy via helplessness and internality on self-management was 0.780 (95% CI [0.373, 1.218]), accounting for 45.29% of the total effect. The indirect effect size of social support via helplessness and internality on self-management was 0.286 (95% CI [0.207, 0.377]), accounting for 57.88% of the total effect. In addition, the differences in effect sizes for

For peer review only - http://bmjopen.bmj.com/site/about/guidelines.xhtml

helplessness and internality in the two models were -0.0797 (95% CI [-0.374, 0.216]) and -0.041 (95% CI [-0.127, 0.043]), respectively. This result indicated that helplessness and internality did not differ significantly in the mediated effect size of the health literacy/social support and self-management relationship model. (Table 4)

Table 4 Bootstrap analysis of multiple mediation effects.

	Effect Size	SE	LLCI	ULCI
X1 (Social Support)				
Total	0.494	0.060	0.375	0.612
Direct	0.208	0.061	0.088	0.328
Total Indirect	0.286	0.043	0.207	0.377
Indirect effect (M1=Helplessness)	0.122	0.030	0.068	0.186
Indirect effect (M2=Internality)	0.163	0.032	0.108	0.230
(C1)	-0.04	0.043	-0.127	0.043
X2 (Health Literacy)				
Total	1.723	0.139	1.449	1.996
Direct	0.942	0.259	0.432	1.453
Total Indirect	0.780	0.217	0.373	1.218
Indirect effect (M1=Helplessness)	0.350	0.119	0.127	0.599
Indirect effect (M2=Internality)	0.430	0.143	0.161	0.720
(C1)	-0.080	0.148	-0.374	0.216

Note: C1=Helplessness minus Internality.

DISCUSSION

For peer review only

Based on the COM-B model, this study jointly explored potential factors influencing self-management in MHD patients at three levels: opportunity, competence, and motivation, and elucidated the pathways among the factors by constructing a theoretical model. Our findings confirm the COM-B model's applicability in health-related behavioral research among MHD patients. First, health literacy/social support, as an important competence and opportunity factor for MHD patients, was positively and negatively associated with internality and helplessness, respectively, and positively associated with self-management. Second, learned helplessness, an important motivational factor influencing individual behavior, and its two major systems, internality and helplessness, were positively and negatively associated with self-management in MHD patients, respectively. Furthermore, our findings suggest that health literacy/social support influences self-management through internality and helplessness as mediating factors.

It is worth noting that internality, the core system of LH, has similarities with self-efficacy in that both can directly or indirectly influence self-management. However, the two are presented in different theoretical contexts, determining the intervention's core. Self-efficacy, derived from Bandura's social learning theory[45], refers to an individual's subjective evaluation of his or her abilities, driven by personal expectations and goals. In this context, the core of self-management interventions is to help patients set reasonable goals and gradually develop their self-confidence. Internality originates from Rotter's locus of control theory[31], which refers to a stable way of attributing the outcome of events internally after individuals

For peer review only - http://bmjopen.bmj.com/site/about/guidelines.xhtml

have learned through experience. The core of its health behavior intervention is to guide patients to rational attribution, reduce helplessness and enhance motivation.

Correlation between health literacy/social support and learned helplessness

This study first verified the correlation between health literacy and the component of learned helplessness (internality/helplessness). According to the knowledge-attutide-practice theory[46], patients with some knowledge of kidney disease and dialysis rated their self-behaviour better and had stronger beliefs about their illness. They could make rational judgments and perceptions when encountering negative disease-related events, accompanied by a lower sense of helplessness, which was verified in this study. Conversely, patients with higher internality are more likely to acquire disease-related knowledge through various means, which is vital for patients' self-management. Those patients who were chronically helpless lacked the motivation to consistently learn self-care knowledge and skills. This point echoes the findings of our previous qualitative study[47]. Therefore, successful renal disease education should provide knowledge and increase motivation. Healthcare professionals should also emphasize patient internality and empowerment to help patients maintain a positive mindset and long-term intrinsic motivation to face their disease.

Next, this study verified the close relationship between internality/helplessness and social support. According to Shahriari[48], social support, as an important external resource, can lead to significant psychological changes in patients. This study reflects

For peer review only - http://bmjopen.bmj.com/site/about/guidelines.xhtml

two critical variables of internality and helplessness. On the one hand, a lack of social support can induce helplessness in patients, mainly depression, social avoidance, and abandonment. On the other hand, adequate social support can help patients expand access to social resources, increase internality in coping with illness, and reduce helplessness, which is similar to the findings of Wang[49] and Wu[50]. It is worth noting that Jansen[51] noted that excessive support given to MHD patients may affect their internality. In this regard, assisting patients to build an appropriate social support environment is essential to enhance their internality and temporarily escape from helplessness.

Relevance of learned helplessness to self-management

According to Seligman, learned helplessness is essential to human behavior[52]. The present study first verified that helplessness is a negative predictor of self-management behaviour, a result similar to other studies[53-55]. Helplessness plays an important cognitive, motivational, and behavioral role in individuals. It can influence patients' attitudes toward illness and treatment and determine their health-related behaviors and persistence[56]. MHD patients feel isolated due to worsening kidney function, complications, and death anxiety. In addition, anxiety, panic, depression, regret, and social isolation are all feelings that contribute to the emotional burden of MHD patients. Many accept their condition with a sense of helplessness—a feeling that whatever they do will not have an impact on the future. Overwhelming helplessness may weaken patients' motivation to engage in treatment.

For peer review only - http://bmjopen.bmj.com/site/about/guidelines.xhtml

This sense of helplessness may also be reinforced by recurrent experiences of uncontrollable symptoms, which may be deeply rooted in the background of living with the situation.

Next, we verified that internality is a positive predictor of self-management, which is consistent with other studies[57 58]. Numerous scholars have argued that control is one of the basic psychological needs of humans, determined by humans' social attributes. Internality determines the degree to which an individual can grasp stressful events. When patients have high internality, they can actively use various resources and establish a more positive way of coping with their illness. In this study, MHD patients with higher internality tended to perceive the existing treatment outcome as a result of their behavior. For example, for adverse events in disease treatment, patients more often attributed them to their lack of effort and believed they could mitigate the effects of adverse events by changing their behaviors.

The mediating role of learned helplessness

The results showed that health literacy/social support could influence the self-management of MHD patients through the mediating role of helplessness and internality, similar to other studies[56 59]. First, health literacy reflects patients' perceptions of illness and treatment and can determine their ability and motivation to take positive steps to improve their health. Patients with higher health literacy have a higher ability to access medical information and communicate with medical staff and are better able to make rational judgments independently when faced with changes in

For peer review only - http://bmjopen.bmj.com/site/about/guidelines.xhtml

their health status, thereby reducing feelings of helplessness. Individuals with a correct understanding of the disease can influence their emotional responses. When MHD patients have more knowledge about kidney disease and health care, their ability to perceive disease deterioration is enhanced, and their sense of control over the disease increases. Based on this, patients can more actively and purposefully engage in their health behavior.

Second, as an essential external resource for individuals, social support can effectively help buffer the harm caused by stressors and provide tangible and intangible support. Most MHD patients have experienced failures in disease control due to the irreversibility of ESKD, when the lack of social support, such as family support and medical support, can induce helplessness[60]. Helpless patients may feel that they cannot change their status through their behaviors. Therefore, they have less internality and gradually show external control tendencies. They tend to attribute the outcome of their behaviors to external conditions (fate, social background, power, etc.) and believe that the result of events is more controlled by external conditions or other forces. They have less control over their behaviors. Most patients' behaviors are characterized by self-abandonment and lack of subjective initiatives, such as social avoidance, reluctance to chat, lack of novelty, and even decreased trust in people around them. Internality, as an important motivational factor, is combined with social support to make patients feel a favorable internal and external environment and resources. The confidence to control the disease outcome is subsequently increased, which can motivate patients to take the initiative to adopt health-related behaviors.

For peer review only - http://bmjopen.bmj.com/site/about/guidelines.xhtml

Implications for future research

The results of this study confirmed that helplessness and internality are important ways for health literacy/social support to influence self-management behaviours. The results help us understand the mechanisms of possible changes in the health-related behaviors of MHD patients and provide theoretical support for future studies. In the future, health care providers can focus on learned helplessness as an intervention target and identify and assess the level of learned helplessness as early as possible while providing health education and medical support for MHD patients. Patients can be empowered by encouraging or rewarding their self-management behaviours to improve their internality and reduce helplessness.

Seligman et al.[61] showed that individual helplessness also triggers a group's helplessness, harming individual behavior. Most MHD patients in China have wardmate groups, and learned helplessness events among peers may impact other patients' behaviors. Future studies may investigate the interaction of learned helplessness in groups on MHD patients' behaviors.

Limitations and future directions

The current study has several limitations that could be addressed in future research. First, we selected dialysis patients from a southern city in China by convenience sampling, which may limit the generalizability of the results. Based on the ethnic diversity of China, future studies could recruit participants from more diverse cultures

For peer review only - http://bmjopen.bmj.com/site/about/guidelines.xhtml

and backgrounds. Second, causality cannot be inferred from the current study, and future longitudinal studies are needed. Third, the reliance on self-reported data may pose a potential risk to the validity of the measurements.

CONCLUSION

The present study further explained the mechanism by which health literacy/social support influences self-management. It reveals the importance of learned helplessness on self-management behavior change in MHD patients. This study's parallel mediator models contribute to developing and improving intervention strategies for MHD patients' health-related behaviors. This suggests that clinical caregivers should pay attention to preventing, identifying, and monitoring patients' learned helplessness when providing health education and medical support to MHD patients. Reducing their helplessness and increasing their internality enhance their motivation and persistence in self-management.

Acknowledgements

We express our heartfelt gratitude to all members who assisted in the data collection and processing and to every MHD patient who participated in this study.

Contributors

For peer review only - http://bmjopen.bmj.com/site/about/guidelines.xhtml

CX contributed to the conceptualization, data curation, formal analysis, and writing the original draft. LL made contributions to the data curation and formal analysis. YL contributed to the writing review and editing.

Funding

This research received grants from the National Natural Science Foundation of China (No.81873806), Major Scientific and Technological Projects in Hunan Province (No.2020SK2085), 2022 Hunan Province Postgraduate Scientific Research Innovation Project (No.12300-150110021), and 2022 Central South University Postgraduate Independent Exploration and Innovation Program (No. 1053320213471).

Competing interests

All authors declare that they have no conflicts of interest.

Patient consent for publication

Obtained.

Provenance and peer review

Not commissioned; externally peer reviewed.

Data availability statement

For peer review only - http://bmjopen.bmj.com/site/about/guidelines.xhtml

The datasets analysed during the current study are not publicly available because they contain information that could compromise research participant consent but are available from the corresponding author on reasonable request.

Figure legends

Figure 1. Theoretical framework based on the COM-B model. HL: Health literacy; LH: Learned helplessness; SS: Social support; SM: Self-management.

Figure 2. Parallel multiple mediator models. A: Health Literacy Model, B: Social Support Model.

Ethics approval

The study was approved by the Ethics Committee of Xiangya Nursing School of Central South University on April 20, 2022 (No. E202255). Informed consent was obtained from all participants.

References

1. Collaboration GCKD. Global, regional, and national burden of chronic kidney disease, 1990–2017: a systematic analysis for the Global Burden of Disease Study 2017. *Lancet* 2020;395(10225):709-33. doi: 10.1016/S0140-6736(20)30045-3
2. O'Hare AM. Dialysis adequacy reconsidered: The person comes first. *Seminars in dialysis* 2020;33(6):486-89. doi: 10.1111/sdi.12938 [published Online First: 2020/11/27]
3. Wang S-Y, Zang X-Y, Fu S-H, et al. Factors related to fatigue in Chinese patients with end-stage renal disease receiving maintenance hemodialysis: a multi-center cross-sectional study. *Renal failure* 2016;38(3):442-50. doi: 10.3109/0886022X.2016.1138819
4. He Y, Lu Y, Yang S, et al. Dietary Plant Protein and Mortality Among Patients Receiving Maintenance Hemodialysis: A Cohort Study. *American journal of kidney diseases : the official journal of the National Kidney Foundation* 2021;78(5):649-57.e1. doi: 10.1053/j.ajkd.2021.03.023 [published Online First: 2021/05/31]

For peer review only - http://bmjopen.bmj.com/site/about/guidelines.xhtml

5. Meng Y, Wu H-t, Niu J-l, et al. Prevalence of depression and anxiety and their predictors among patients undergoing maintenance hemodialysis in Northern China: a cross-sectional study. *Ren Fail* 2022;44(1):933-44. doi: 10.1080/0886022X.2022.2077761
6. Brioni E, Delli Zotti G, Apuzzo L, et al. Self-efficacy and self-management in patients in hemodialysis: a narrative review of multidisciplinary theories. *G Ital Nefrol* 2021;38(3):2021-vol3. Italian.
7. Griva K, Nandakumar M, Jo-an HN, et al. Hemodialysis self-management intervention randomized trial (HED-SMART): a practical low-intensity intervention to improve adherence and clinical markers in patients receiving hemodialysis. *American Journal of Kidney Diseases* 2018;71(3):371-81. doi: 10.1053/j.ajkd.2017.09.014
8. Curtin RB, Sitter DC, Schatell D, et al. Self-management, knowledge, and functioning and well-being of patients on hemodialysis. *Nephrology Nursing Journal* 2004;31(4):378-86.
9. Tovazzi ME, Mazzoni V. Personal paths of fluid restriction in patients on hemodialysis. *Nephrology Nursing Journal* 2012;39(3):207-15.
10. Li H, Cao YD, Jiang YF, et al. Reliability and validity tests of Hemodialysis Self-Management Instrument (in Chinese). *Chinese Journal of Nursing* 2015;50(11):1392-95. doi: 10.3761/j.issn.0254-1769.2015.11.024
11. Joboshi H, Oka M. Effectiveness of an educational intervention (the Encourage Autonomous Self-Enrichment Program) in patients with chronic kidney disease: a randomized controlled trial. *International journal of nursing studies* 2017;67:51-58. doi: 10.1016/j.ijnurstu.2016.11.008
12. Nutbeam D. The evolving concept of health literacy. *Social science medicine* 2008;67(12):2072-78. doi: 10.1016/j.socscimed.2008.09.050
13. Lopez-Vargas PA, Tong A, Howell M, et al. Educational interventions for patients with CKD: a systematic review. *American Journal of Kidney Diseases* 2016;68(3):353-70. doi: 10.1053/j.ajkd.2016.01.022
14. Devraj R, Borrego M, Vilay AM, et al. Relationship between health literacy and kidney function. *Nephrology* 2015;20(5):360-67. doi: 10.1111/nep.12425
15. Wu SFV, Hsieh NC, Lin LJ, et al. Prediction of self‐care behaviour on the basis of knowledge about chronic kidney disease using self‐efficacy as a mediator. *Journal of clinical nursing* 2016;25(17-18):2609-18. doi: 10.1111/jocn.13305
16. Hu C, Lim P, Chang T. The effectiveness of case management on self-care and quality of life in chronic kidney disease patients. *The Journal of Health Science* 2012;14(1):13-28.
17. Tifferet S. Gender differences in social support on social network sites: A meta-analysis. *Cyberpsychology, behavior, social networking* 2020;23(4):199-209. doi: 10.1089/cyber.2019.0516
18. Cullen FT. Social support as an organizing concept for criminology: Presidential address to the Academy of Criminal Justice Sciences. *Justice Quarterly* 1994;11(4):527-59. doi: Cullen FT. Social support as an organizing concept for criminology: Presidential address to the Academy of Criminal Justice Sciences. Justice Quarterly 1994;11(4):527-59.
19. Cardol CK, Boslooper-Meulenbelt K, van Middendorp H, et al. Psychosocial barriers and facilitators for adherence to a healthy lifestyle among patients with chronic kidney disease: A focus group study. *BMC nephrology* 2022;23(1):1-16. doi: 10.1186/s12882-022-02837-0
20. Neri L, Martini A, Andreucci VE, et al. Regimen complexity and prescription adherence in dialysis patients. *American journal of nephrology* 2011;34(1):71-76. doi: 10.1159/000328391

For peer review only - http://bmjopen.bmj.com/site/about/guidelines.xhtml

21. Kauric-Klein Z. Predictors of nonadherence with blood pressure regimens in hemodialysis. *Patient preference adherence* 2013;7:973. doi: 10.2147/PPA.S45369
22. Song Y-y, Chen L, Wang W-x, et al. Social Support, Sense of Coherence, and Self-Management among Hemodialysis Patients. *Western Journal of Nursing Research* 2022;44(4):367-74. doi: 10.1177/0193945921996648
23. Been‐Dahmen JM, Grijpma JW, Ista E, et al. Self‐management challenges and support needs among kidney transplant recipients: A qualitative study. *Journal of advanced nursing* 2018;74(10):2393-405. doi: 10.1111/jan.13730
24. Seligman ME. Learned helplessness as a model of depression. Comment and integration. *Journal of abnormal psychology* 1978;87(1):165-79. doi: 10.1037//0021-843x.87.1.49
25. Camacho EM, Verstappen SM, Chipping J, et al. Learned helplessness predicts functional disability, pain and fatigue in patients with recent-onset inflammatory polyarthritis. *Rheumatology (Oxford, England)* 2013;52(7):1233-8. doi: 10.1093/rheumatology/kes434 [published Online First: 2013/02/21]
26. Crielaard L, Nicolaou M, Sawyer A, et al. Understanding the impact of exposure to adverse socioeconomic conditions on chronic stress from a complexity science perspective. *BMC medicine* 2021;19(1):242. doi: 10.1186/s12916-021-02106-1 [published Online First: 2021/10/13]
27. Peterson C, Seligman ME, Vaillant GE. Pessimistic explanatory style is a risk factor for physical illness: a thirty-five-year longitudinal study. *Journal of personality and social psychology* 1988;55(1):23-7. doi: 10.1037//0022-3514.55.1.23 [published Online First: 1988/07/01]
28. Yu-Ying C, Li-Ya T. Applying Swanson's Caring Theory to a Patient With End Renal Stage Disease Experiencing Hopelessness. *Journal of Nursing* 2021;68(2):99-106. doi: 10.6224/JN.202104_68(2).13
29. Kurita N, Wakita T, Fujimoto S, et al. Hopelessness and Depression Predict Sarcopenia in Advanced CKD and Dialysis: A Multicenter Cohort Study. *The journal of nutrition, health & aging* 2021;25(5):593-99. doi: 10.1007/s12603-020-1556-4 [published Online First: 2021/05/06]
30. DeVellis R, Callahan L. A brief measure of helplessness in rheumatic disease: the helplessness subscale of the Rheumatology Attitudes Index. *The Journal of rheumatology* 1993;20(5):866-69.
31. Rotter JB. Generalized expectancies for internal versus external control of reinforcement. *Psychol Monogr Gen Appl* 2010;80:1–28.
32. Khalil AA, Abed MA. Perceived social support is a partial mediator of the relationship between depressive symptoms and quality of life in patients receiving hemodialysis. *Archives of Psychiatric Nursing* 2014;28(2):114-18. doi: 10.1016/j.apnu.2013.11.007
33. Bayuo J, Bristowe K, Harding R, et al. "Managing uncertainty": Experiences of family members of burn patients from injury occurrence to the end-of-life period. *Burns : journal of the International Society for Burn Injuries* 2021;47(7):1647-55. doi: 10.1016/j.burns.2021.02.009 [published Online First: 2021/04/10]
34. Smallheer BA, Vollman M, Dietrich MS. Learned helplessness and depressive symptoms following myocardial infarction. *Clinical Nursing Research* 2018;27(5):597-616. doi: 10.1177/1054773816689752
35. Kail BL, Carr DC. Successful aging in the context of the disablement process: Working and volunteering as moderators on the association between chronic conditions and subsequent

For peer review only - http://bmjopen.bmj.com/site/about/guidelines.xhtml

functional limitations. *The Journals of Gerontology: Series B* 2017;72(2):340-50. doi: 10.1093/geronb/gbw060

36. Michie S, Van Stralen MM, West R. The behaviour change wheel: a new method for characterising and designing behaviour change interventions. *Implementation science* 2011;6(1):1-12. doi: 10.1186/1748-5908-6-42

37. Murtagh EM, Barnes AT, McMullen J, et al. Mothers and teenage daughters walking to health: using the behaviour change wheel to develop an intervention to improve adolescent girls' physical activity. *Public health* 2018;158:37-46. doi: 10.1016/j.puhe.2018.01.012

38. Li H. Research on the Condition and Influencing Factors ofHemodialysis Patients' Self Management (in Chinese). Peking Union Medical College, 2011.

39. Xiao SY. The theoretical basis and research application of the Social Support Rating Scale (in Chinese). *Journal of Clinical Psychiatry* 1994;99(04):98–100. doi: CNKI:SUN:LCJS.0.1994-02-019

40. Jiang H, Wang L, Zhang Q, et al. Family functioning, marital satisfaction and social support in hemodialysis patients and their spouses. *Stress Health* 2015;31(2):166-74. doi: 10.1002/smi.2541

41. Song YJ. Construction and testing of a self-management scale for hemodialysis patients (in Chinese). Kaohsiung Medical University, 2009.

42. Li H, Cao YD, Jiang YF, et al. Introduction and reliability testing of a self-management scale for hemodialysis patients (in Chinese). *Chin J Nurs* 2015;50(11):1392-95. doi: 10.3761/j.issn.0254-1769.2015.11.024

43. Nicassio PM, Wallston KA, Callahan LF, et al. The measurement of helplessness in rheumatoid arthritis. The development of the arthritis helplessness index. *J Rheumatol* 1985;12(3):462-67. doi: 10.1007/BF02031627

44. Xie C, Zhou L, Sun C, et al. The cross - cultural adaptation and psychometric evaluation of a learned helplessness scale for maintenance hemodialysis patients in China (LHS - MHD - C). *Res Nurs Health* 2023;Advance online publication doi: 3.80599.net/10.1002/nur.22298

45. Bandura A. The explanatory and predictive scope of self-efficacy theory. *J Soc Clin Psychol* 1986;4(3):359-73. doi: 10.1521/jscp.1986.4.3.359

46. Oshima T, Siah KT, Kim YS, et al. Knowledge, attitude, and practice survey of gastroparesis in Asia by Asian Neurogastroenterology and Motility Association. *Journal of neurogastroenterology motility* 2021;27(1):46. doi: 10.5056/jnm20117

47. Xie C, Li L, Li Y. “Alive Day is the Day”: A Qualitative Study of Experiences of Learned Helplessness in Maintenance Haemodialysis Patients. *Risk Manag Healthc Policy* 2023:231-45. doi: 10.2147/RMHP.S401205

48. Shahriari M, Alimohammadi N, Ahmadi M. Effects of a family centered program on perceived social support in patients with congestive heart failure: An interventional study. *Journal of Education Health Promotion International* 2016(Vol. 5):1-6. doi: 10.4103/2277-9531.184559

49. Wang XN, Zhang H, Xie ZY. Analysis of the current status of self-concealment in maintenance hemodialysis patients and its correlation and influencing factors with disease stigma and social support (in Chinese). *Shandong Medical Journal* 2020;60(17):84-86. doi: 10. 3969 / j. issn. 1002-266X. 2020. 17. 025

50. Wu H. Self-perceived burden and uncertainty in illness in maintenance hemodialysis patients and related research on social support (in Chinese). Southwest Medical University, 2020.

For peer review only - http://bmjopen.bmj.com/site/about/guidelines.xhtml

51. Jansen DL, Rijken M, Kaptein AA, et al. The role of social support in dialysis patients' feelings of autonomy and self-esteem: Is support more beneficial for patients with specific illness perceptions? *Families, Systems, Health* 2014;32(3):313. doi: 10.1037/fsh0000028

52. Seligman M, Weiss J, Weinraub M, et al. Coping behavior: learned helplessness, physiological change and learned inactivity. *Behaviour research therapy* 1980;18(5):459-512. doi: 10.1016/0005-7967(80)90011-x

53. Neuvonen E, Lehtisalo J, Solomon A, et al. Psychosocial determinants for adherence to a healthy lifestyle and intervention participation in the FINGER trial: an exploratory analysis of a randomised clinical trial. *Aging clinical and experimental research* 2022;34(8):1793-805. doi: 10.1007/s40520-022-02088-x [published Online First: 2022/02/20]

54. Sheridan N, Kenealy T, Salmon E, et al. Helplessness, self blame and faith may impact on self management in COPD: a qualitative study. *Primary Care Respiratory Journal* 2011;20(3):307-14. doi: 10.4104/pcrj.2011.00035

55. Omachi TA, Katz PP, Yelin EH, et al. The COPD Helplessness Index: a new tool to measure factors affecting patient self-management. *Chest* 2010;137(4):823-30. doi: 10.1378/chest.09-0764 [published Online First: 2009/10/20]

56. Wang LM, Chiou CP. Effectiveness of interactive multimedia CD on self-care and powerlessness in hemodialysis patients. *Journal of Nursing Research* 2011;19(2):102-11. doi: 10.1097/JNR.0b013e31821b0eff

57. Fan JL, Kong Y, Shi SH, et al. Correlation Study of Health Locus of Control and Self-management Behaviors in Patients Underwent Hemodialysis (in Chinese). *Nursing Journal of Chinese People's Liberation Army* 2016;33(9):23-27. doi: 10.3969/j.issn.1008-9993.2016.09.005

58. Lael-Monfared E, Tehrani H, Teiho Z, et al. The study of eye care behaviors in patients with type 2 diabetes. *Journal of Diabetes Metabolic Disorders* 2020;19(1):257-63. doi: 10.1007/s40200-020-00499-z

59. Chen CC, Li HP, Liu HQ, et al. A study on the relationship among self - management behavior, family function, locus of control among maintenance hemodialysis patient (in Chinese). *Chinese Journal of health education* 2020;36(11):1053. doi: 10.16168/j.cnki.issn.1002-9982.2020.11.020

60. Kiajamali M, Hosseini M, Estebsari F, et al. Correlation between social support, self-efficacy and health-promoting behavior in hemodialysis patients hospitalized in Karaj in 2015. *Electronic Physician* 2017;9(7):4820. doi: 10.19082/4820

61. Abramson LY, Seligman ME, Teasdale JD. Learned helplessness in humans: critique and reformulation. *Journal of abnormal psychology* 1978;87(1):49. doi: 10.1037//0021-843x.87.1.49

For peer review only - http://bmjopen.bmj.com/site/about/guidelines.xhtml

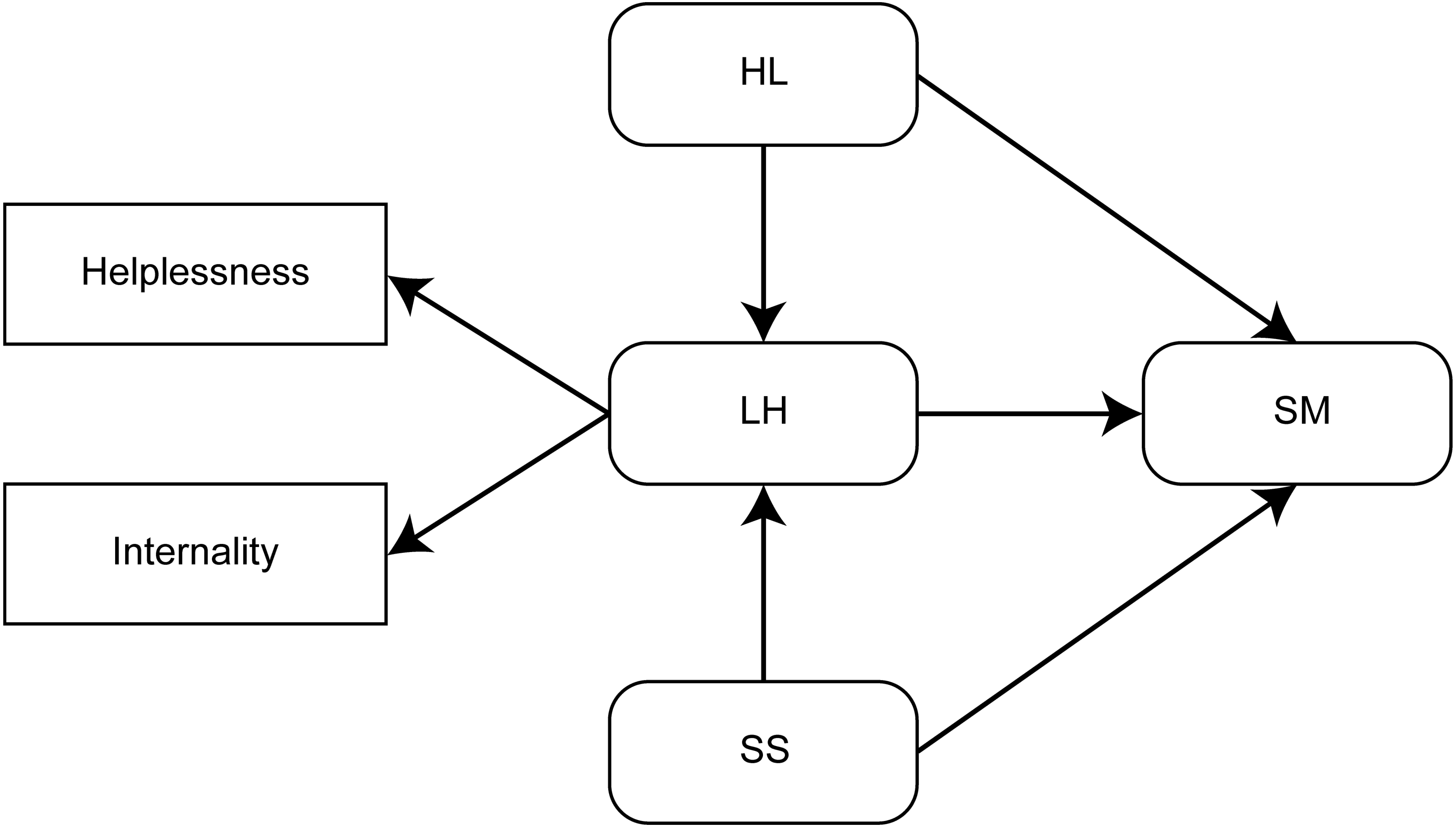
HL
Helplessness
LH
SM
Internality
SS

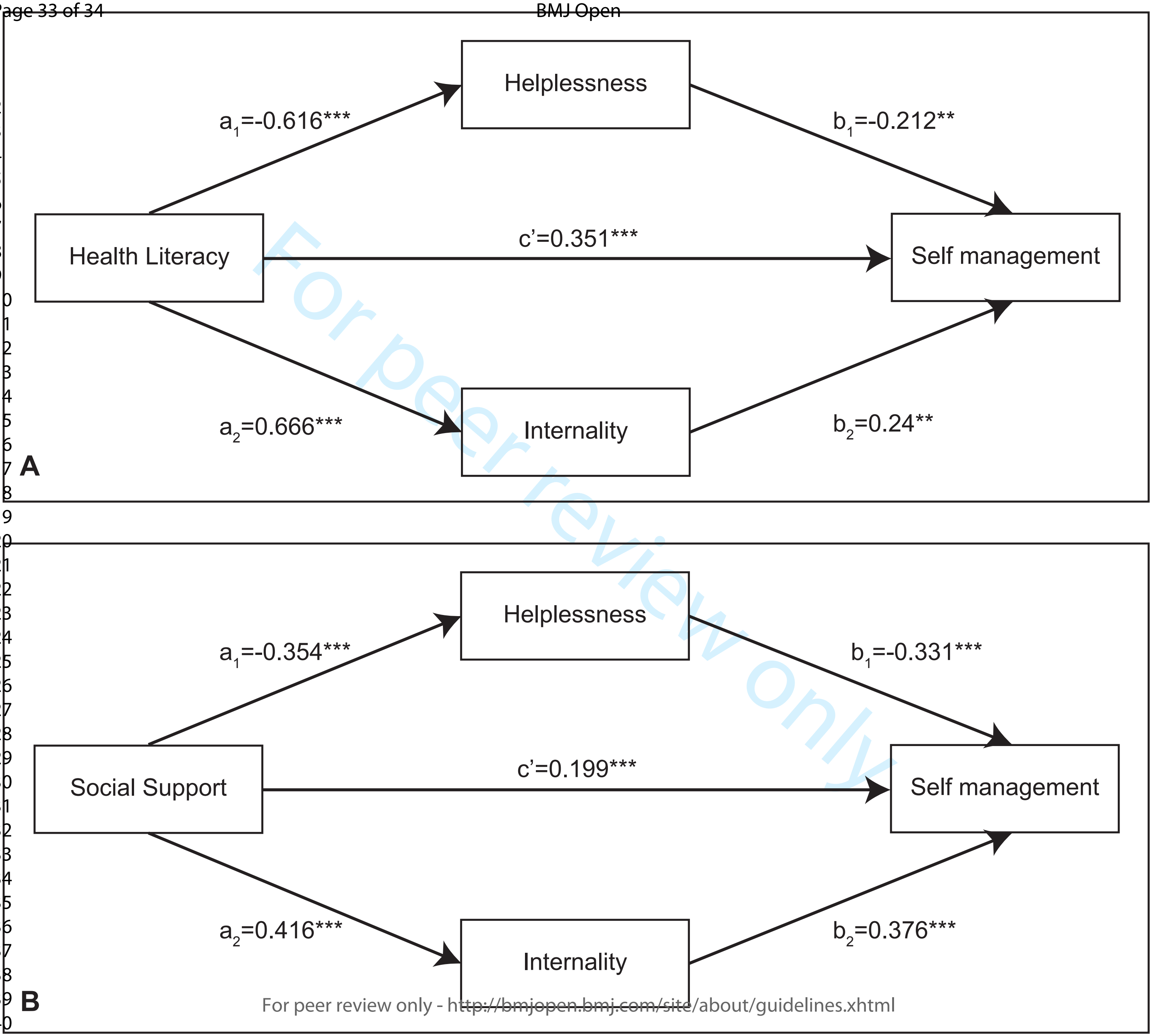

For peer review only

Supplementary File 1

STROBE 2007 (v4) Statement—Checklist of items that should be included in reports of *cross-sectional studies*

Section/Topic	Item #	Recommendation	Reported on page #
**Title and abstract**	1	(*a*) Indicate the study's design with a commonly used term in the title or the abstract	P1
		(*b*) Provide in the abstract an informative and balanced summary of what was done and what was found	P1-2
**Introduction**			
Background/rationale	2	Explain the scientific background and rationale for the investigation being reported	P3-7
Objectives	3	State specific objectives, including any prespecified hypotheses	P8
**Methods**			
Study design	4	Present key elements of study design early in the paper	P8
Setting	5	Describe the setting, locations, and relevant dates, including periods of recruitment, exposure, follow-up, and data collection	P8-9
Participants	6	(*a*) Give the eligibility criteria, and the sources and methods of selection of participants	P8-9
Variables	7	Clearly define all outcomes, exposures, predictors, potential confounders, and effect modifiers. Give diagnostic criteria, if applicable	P9-11
Data sources/ measurement	8*	For each variable of interest, give sources of data and details of methods of assessment (measurement). Describe comparability of assessment methods if there is more than one group	P9-11
Bias	9	Describe any efforts to address potential sources of bias	P11
Study size	10	Explain how the study size was arrived at	P11
Quantitative variables	11	Explain how quantitative variables were handled in the analyses. If applicable, describe which groupings were chosen and why	P11
Statistical methods	12	(*a*) Describe all statistical methods, including those used to control for confounding	P13
		(*b*) Describe any methods used to examine subgroups and interactions	P12-13
		(*c*) Explain how missing data were addressed	P12
		(*d*) If applicable, describe analytical methods taking account of sampling strategy	P9
		(*e*) Describe any sensitivity analyses	N/A

For peer review only

**Results**			
Participants	13*	(a) Report numbers of individuals at each stage of study—eg numbers potentially eligible, examined for eligibility, confirmed eligible, included in the study, completing follow-up, and analysed	N/A
		(b) Give reasons for non-participation at each stage	N/A
		(c) Consider use of a flow diagram	N/A
Descriptive data	14*	(a) Give characteristics of study participants (eg demographic, clinical, social) and information on exposures and potential confounders	P13-14
		(b) Indicate number of participants with missing data for each variable of interest	N/A
Outcome data	15*	Report numbers of outcome events or summary measures	P15
Main results	16	(*a*) Give unadjusted estimates and, if applicable, confounder-adjusted estimates and their precision (eg, 95% confidence interval). Make clear which confounders were adjusted for and why they were included	P13
		(*b*) Report category boundaries when continuous variables were categorized	N/A
		(*c*) If relevant, consider translating estimates of relative risk into absolute risk for a meaningful time period	N/A
Other analyses	17	Report other analyses done—eg analyses of subgroups and interactions, and sensitivity analyses	N/A
**Discussion**			
Key results	18	Summarise key results with reference to study objectives	P18
Limitations	19	Discuss limitations of the study, taking into account sources of potential bias or imprecision. Discuss both direction and magnitude of any potential bias	P23
Interpretation	20	Give a cautious overall interpretation of results considering objectives, limitations, multiplicity of analyses, results from similar studies, and other relevant evidence	P18-22
Generalisability	21	Discuss the generalisability (external validity) of the study results	P23
**Other information**			
Funding	22	Give the source of funding and the role of the funders for the present study and, if applicable, for the original study on which the present article is based	P24

*Give information separately for cases and controls in case-control studies and, if applicable, for exposed and unexposed groups in cohort and cross-sectional studies.

Note: An Explanation and Elaboration article discusses each checklist item and gives methodological background and published examples of transparent reporting. The STROBE checklist is best used in conjunction with this article (freely available on the Web sites of PLoS Medicine at http://www.plosmedicine.org/, Annals of Internal Medicine at http://www.annals.org/, and Epidemiology at http://www.epidem.com/). Information on the STROBE Initiative is available at www.strobe-statement.org.

For peer review only

BMJ Open

The mediating role of learned helplessness's components in the association between health literacy/social support and self-management among maintenance hemodialysis patients in Changsha, China: a cross-sectional study

Journal:	*BMJ Open*
Manuscript ID	bmjopen-2022-068601.R2
Article Type:	Original research
Date Submitted by the Author:	16-Jun-2023
Complete List of Authors:	Xie, Chunyan; The Second Xiangya Hospital of Central South University, Clinical Nursing Teaching and Research Section; Central South University, Xiangya Nursing School<br>Li, Li; The Second Xiangya Hospital of Central South University, Clinical Nursing Teaching and Research Section; Xinjiang Medical University Affiliated First Hospital, Department of Urology<br>Zhou, Lin; The Second Xiangya Hospital of Central South University, Clinical Nursing Teaching and Research Section; The Second Xiangya Hospital of Central South University, Blood Purification Center, Department of Nephrology<br>Sun, Cuifang; The Second Xiangya Hospital of Central South University, Clinical Nursing Teaching and Research Section; The Second Xiangya Hospital of Central South University, Blood Purification Center, Department of Nephrology<br>Zhang, Yini; The Second Xiangya Hospital of Central South University, Clinical Nursing Teaching and Research Section; The Second Xiangya Hospital of Central South University, Blood Purification Center, Department of Nephrology<br>Li, Ya-Min; The Second Xiangya Hospital of Central South University, Clinical Nursing Teaching and Research Section
**Primary Subject Heading**:	Mental health
Secondary Subject Heading:	Nursing, Mental health
Keywords:	MENTAL HEALTH, Dialysis < NEPHROLOGY, Change management < HEALTH SERVICES ADMINISTRATION & MANAGEMENT

SCHOLARONE™ Manuscripts

I, the Submitting Author has the right to grant and does grant on behalf of all authors of the Work (as defined in the below author licence), an exclusive licence and/or a non-exclusive licence for contributions from authors who are: i) UK Crown employees; ii) where BMJ has agreed a CC-BY licence shall apply, and/or iii) in accordance with the terms applicable for US Federal Government officers or employees acting as part of their official duties; on a worldwide, perpetual, irrevocable, royalty-free basis to BMJ Publishing Group Ltd ("BMJ") its licensees and where the relevant Journal is co-owned by BMJ to the co-owners of the Journal, to publish the Work in this journal and any other BMJ products and to exploit all rights, as set out in our licence.

The Submitting Author accepts and understands that any supply made under these terms is made by BMJ to the Submitting Author unless you are acting as an employee on behalf of your employer or a postgraduate student of an affiliated institution which is paying any applicable article publishing charge ("APC") for Open Access articles. Where the Submitting Author wishes to make the Work available on an Open Access basis (and intends to pay the relevant APC), the terms of reuse of such Open Access shall be governed by a Creative Commons licence – details of these licences and which Creative Commons licence will apply to this Work are set out in our licence referred to above.

Other than as permitted in any relevant BMJ Author's Self Archiving Policies, I confirm this Work has not been accepted for publication elsewhere, is not being considered for publication elsewhere and does not duplicate material already published. I confirm all authors consent to publication of this Work and authorise the granting of this licence.

For peer review only

The mediating role of learned helplessness's components in the association between health literacy/social support and self-management among maintenance hemodialysis patients in Changsha, China: a cross-sectional study

Chunyan Xie[1, 2, *], Li Li[1, 3, *], Lin Zhou[1, 4], Cuifang Sun[1, 4], Yini Zhang[1, 4], Yamin Li[1]

[1] Clinical Nursing Teaching and Research Section, The Second Xiangya Hospital, Central South University, Changsha 410011, Hunan, China

[2] XiangYa Nursing School, Central South University, Changsha 410013, Hunan, China

[3] Department of Urology, the First Affiliated Hospital of Xinjiang Medical University, Urumqi 830011, Xinjiang, China

[4] Blood Purification Center, Department of Nephrology, The Second Xiangya Hospital, Central South University, Changsha 410011, Hunan, China

* Chunyan Xie, Li Li are contributed equally to this article.

Correspondence: Yamin Li, The Second Xiangya Hospital, Central South University, Changsha 410011, Hunan, China, Email aminny@csu.edu.cn

Abstract

Objectives: To explore the multiple mediating roles of the learned helplessness's core system in the relationship between health literacy/social support and self-management.

For peer review only - http://bmjopen.bmj.com/site/about/guidelines.xhtml

Design: Cross-sectional survey design.

Setting: Changsha, China.

Participants: 239 Chinese maintenance hemodialysis (MHD) patients.

Methods: Two multiple mediator models were constructed based on the COM-B model. A total of 239 Chinese MHD patients participated in a cross-sectional study, which included surveys on the Learned Helplessness Scale for MHD patients, Dialysis Knowledge Questionnaire, Social Support Scale, and Self-Management Scale for Hemodialysis. The PROCESS macro in SPSS was used for mediated effects analysis.

Results: Helplessness and internality partially mediated the relationship between health literacy/social support and self-management [(β = -0.212, $P < 0.01$; β = 0.240, $P < 0.01$)/(β = -0.331, $P < 0.001$; β = 0.376, $P < 0.001$)]. The mediation effect size was 0.780 (95% CI [0.373, 1.218]) in the health literacy model, accounting for 45.29% of the total effect, and 0.286 (95% CI [0.207, 0.377]) in the social support model, accounting for 57.88% of the total effect. The differences in effect sizes for helplessness and internality in the two models were -0.080 (95% CI [-0.374, 0.216]) and -0.041 (95% CI [-0.127, 0.043]), respectively.

Conclusion: Health literacy/social support directly affects MHD patients' self-management and indirectly affects it by changing learned helplessness, such as increasing internality while reducing helplessness.

Keywords: learned helplessness, self-management, health literacy, social support, multiple mediations

For peer review only - http://bmjopen.bmj.com/site/about/guidelines.xhtml

Strengths and limitations of this study:

1. The PROCESS macro in SPSS was used to verify the potential mediating effects of learned helplessness's components on the relationship between health literacy/social support and self-management in Chinese maintenance hemodialysis patients.

2. A culturally adapted and validated Chinese version of the Learned Helplessness Scale for Maintenance Hemodialysis Patients was used.

3. Based on the ethnic diversity of China, participants could be recruited from a more diverse range of cultures and backgrounds.

4. Causality cannot be inferred from the current study, and future longitudinal studies are needed.

5. The self-reported data may pose a potential risk to the validity of the measurements.

BACKGROUND

By 2017, approximately 700 million patients worldwide had chronic kidney disease (CKD), with China and India accounting for a significant portion (132 and 115 million, respectively) .[1] End-stage kidney disease (ESKD) refers to a complete or irreversible decline in renal function. Maintenance hemodialysis (MHD) is the most widely used alternative treatment in clinical practice.[2,3] MHD is defined as regular hemodialysis patients receiving for more than three months with a frequency of 2-3 times per week.[4] In China, most ESKD patients are treated with MHD.[5]

For peer review only - http://bmjopen.bmj.com/site/about/guidelines.xhtml

For peer review only

Although MHD can prolong their lives, it cannot completely replace renal function. Patients must also slow their disease progression through self-management, such as long-term medications, restricted diet, fluid intake, regular exercise, and arteriovenous fistula care.[6,7] Curtin et al.[8] showed that self-management behaviours positively impacted MHD patients' overall health status. However, a large number of studies point to the generally unsatisfactory self-management status of MHD patients. For example, less than 40% of them can strictly follow the doctor's advice to control their diet and water.[9] The overall level of Chinese MHD patients' self-management is low, with a score of 45.21 ± 13.44 (total scale score of 80).[10] Therefore, it has become a priority for healthcare staff to investigate the controllable factors influencing MHD patients' self-management to improve their health status and quality of life.

Health literacy/social support and self-management

A growing body of research has focused on how to improve MHD patients' self-management behaviours. Health literacy is an important influencing factor for improving self-management behaviours,[11] which reflects the degree of patients' awareness of the disease and determines their motivation and ability to acquire, understand, and use knowledge to improve their health.[12] Notably, nursing education aims to improve patients' perceptions of disease and overall health literacy to induce changes in their behaviors.[13] When patients are more aware of their disease and health, they become aware of the importance of self-management and are

For peer review only - http://bmjopen.bmj.com/site/about/guidelines.xhtml

more likely to adopt healthy behaviors, as confirmed by more studies. For example, Devraj et al.[14] showed that health literacy in CKD patients was positively associated with self-management, and a randomized controlled trial in Japan confirmed similar results.[11] Wu et al.[15] found that health literacy could influence CKD patients' self-management through the mediating role of self-efficacy, which is consistent with Hu et al..[16]

Second, social support is a factor that significantly impacts the treatment of most patients with chronic diseases.[17] It is a multidimensional concept that refers to the spiritual and material support given to individuals by various parties, such as family, relatives, friends, and social organizations.[18] Adequate social support can reduce MHD patients' financial or psychological burdens and enable them to manage life better.[19] Several studies[20,21] have shown that the lack of family support in MHD patients is followed by poor medication adherence. Song et al.[22] used a structural equation modelling approach to confirm that social support directly affects MHD patients' self-management and, together with the sense of consistency, explained 69% of the variance in self-management behavior. Been-Dahmen et al.[23] noted through qualitative interviews that family, friends, professionals, and colleagues also provide essential emotional support to kidney transplant patients. Thus, social support is crucial for MHD patients to achieve their health goals.

Although previous studies have confirmed the relationship between health literacy, social support, and self-management, most studies have overlooked possible potential pathways for this relationship. Therefore, the study was dedicated to

For peer review only - http://bmjopen.bmj.com/site/about/guidelines.xhtml

exploring the multiple potential possibilities between health literacy/social support and self-management.

Learned helplessness (LH)

Seligman first observed LH in 1967.[24] LH is described as a psychological state of powerlessness or self-abandonment when the individual perceives a disconnection between behavior and outcome, manifested by cognitive deficits, decreased motivation, and emotional maladjustment.[25,26] LH is often used to explain the phenomenon of diminished or absent motivation in individuals and extends to health-related problems.[27] For example, helpless patients adhere significantly less to regular treatment and seem to have more difficulty carrying out physicians' recommendations for diet, water, and exercise.[28,29] LH was divided into two core components. Helplessness is the individual's perception that their behavior cannot impact the outcome.[30] Internality refers to the individual's perception that they believe that they are the most dominant force dominating their life.[31]

It has been suggested that LH is associated with low social support and health literacy. First, adequate social support gives patients more tangible resources for life and intangible resources for spirituality, which can buffer the multiple negative emotions associated with illness.[32] Those with high social support have access to disease-related knowledge through multiple channels to reduce helplessness when coping with the disease. Notably, family support always plays a vital role in reducing patients' LH.[33] Smallheer et al.[34] suggested that emotional support from family

For peer review only - http://bmjopen.bmj.com/site/about/guidelines.xhtml

and friends, access to disease-related knowledge, and trust and support with fellow patients and healthcare staff can help prevent patients' helplessness. In addition, it has been suggested that social support and health literacy can help maintain physical and mental harmony and influence helplessness by integrating them.[35]

Theoretical basis

The Capacity, Opportunity, Motivation - Behavior (COM-B) model, proposed by Michie, provides a framework for understanding individual behavior change.[36] According to this model, although people with MHD are willing to engage in self-management, they give little action, which may be closely related to their ability, opportunity, and motivation. Capacity refers to individuals' knowledge and skills to engage in behavior change.[37] Opportunity refers to the external factors that promote behavior change.[37] Motivation refers to the beliefs that guide an individual's behavior change.[37] In this study, the good self-management exhibited by the patients first required a certain knowledge and skills of kidney disease care, i.e., dialysis-related health literacy. At the opportunity level, social support from family members, friends, and healthcare providers gives emotional and material security to patients. However, chronic LH can undermine patients' motivation to engage in self-management and affect their proactive health behaviors.

After a literature review and theoretical analysis, this study finally developed a mediating model with two core systems of LH as motivational factors, using social

For peer review only - http://bmjopen.bmj.com/site/about/guidelines.xhtml

support as an opportunity factor, health literacy as a competence factor, and self-management as a dependent variable. (As shown in Supplemental Figure)

The current study

There may be a relationship between health literacy/social support, LH, and self-management behaviours. However, the underlying mechanisms between these relationships remain to be further identified. In this study, we developed two multiple mediator models based on the COM-B model to help us understand the role of LH in forming and changing health behaviors. To test this, we made the following hypotheses.

Hypothesis 1 (H1): Health literacy/social support can influence two dimensions of LH (helplessness, internality).

Hypothesis 2 (H2): Two dimensions of LH (helplessness, internality) predict self-management.

Hypothesis 3 (H3): Two dimensions of LH (helplessness, internality) parallelly mediate the relationship between health literacy/social support and self-management.

METHODS

Study design and participants

This is a cross-sectional study to investigate the association between health literacy/social support and self-management through the mediating role of the component of lean helplessness. This study followed the Strengthening the Reporting

For peer review only - http://bmjopen.bmj.com/site/about/guidelines.xhtml

of Observational Studies in Epidemiology Checklist for cross-sectional studies (STROBE 2007, Supplementary File 1) guidelines to ensure the quality and specification of study reporting.

We used convenience sampling to select patients from a comprehensive Grade 3A hospital in Changsha, Hunan Province, China, who met the following criteria: (i) being treated with hemodialysis for ≥ 3 months, (ii) being informed and agreeing to participate in this study, and (iii) being conscious, being literate and having communication abilities. We excluded patients with mental illness, cognitive dysfunction, or serious complications. The purpose and significance of this study were clarified to the participants before the survey to obtain informed consent. The questionnaires were administered 1 hour after the start or 1 hour before the end of dialysis because the patients' blood circulation was more stable. Each participant took approximately 10-15 minutes to complete the questionnaire.

Measures

Health Literacy

We used the Dialysis Knowledge Questionnaire developed by Curtin[8] to measure MHD patients' health literacy. The questionnaire was divided into five dimensions after cross-cultural adaptation by Li.[38] It includes anemia, diet and medication, kidney function, dialysis, and rehabilitation, with 24 items. The patients were asked to judge whether the items' descriptions were true, scoring 1 for a correct answer and 0 for an incorrect/unknown answer. The total score was 24. The higher the score, the

For peer review only - http://bmjopen.bmj.com/site/about/guidelines.xhtml

higher the patient's knowledge of dialysis. Its content validity index (CVI) was 0.94, and the Cronbach's alpha coefficient was 0.701.

Social support

The Social Support Rating Scale (SSRS) developed by Xiao[39] was selected for this study. The scale consists of ten items involving three dimensions: objective support, subjective support, and support utilization. Items 1-4 and 8-10 were scored on a 4-point Likert scale. Item 5 has five subitems, each of which has four options from "none" to "fully support," scoring 1-4 points. The scores of items 6 and 7 are calculated according to the number of support sources. The total score ranges from 12 to 66, with higher scores indicating higher levels of social support. The Cronbach's alpha coefficient for the total scale was 0.896. It has been used in Chinese renal dialysis patients.[40]

Self-management

The Self-Management Scale for Hemodialysis (SMSH), developed by Song[41] in 2009, was used to measure MHD patients' self-management behaviors in this study. It has 20 items and is divided into four dimensions: problem solving, performing self-care, partnership, and emotional processing. The scale was scored on a 4-point Likert scale, with scores 1-4 representing the frequency of the behavior, i.e., never, occasionally, often, and always, respectively. The total score was 20-80. The higher

For peer review only

For peer review only - http://bmjopen.bmj.com/site/about/guidelines.xhtml

the score, the better the patient's self-management behaviour. It was revised by Chinese Li, and its Cronbach's alpha coefficient was 0.813.[42]

LH

There is no special scale for MHD patients' LH. Considering this limitation, we formed a tool called LHS-MHD--C based on Nicassio's AHI scale.[43] Under his authorization, we conducted cross-cultural adaptation, psychological measurement, and evaluation. The LHS-MHD-C has 11 items and was classified into two dimensions (helplessness/internality) based on the results of exploratory factor analysis (EFA) and confirmatory factor analysis (CFA), in line with the LH Theory. It used a five-point Likert scale with response options ranging from 1 = strongly disagree to 5 = strongly agree. Six items in internality are reverse scored. The higher the total score, the more severe the LH. In our previous study, the scale showed good internal consistency [(Cronbach's α of 0.759), retest reliability (n=30, intragroup correlation=0.772), and split-half reliability [0.774].[44]

Statistical analysis

According to numerous similar studies, the sample size of this study was initially determined by the observed variables of the theoretical model (i.e., the ratio of sample size to observed variables should be at least between 10:1 and 15:1).[45] Additionally, the formula[46] for proportional variables is $n=[Z^2P(1-P)]/d^2$, where Z is the statistic at a 95% confidence level (1.96), P is the proportion of the target population (0.1081),

For peer review only - http://bmjopen.bmj.com/site/about/guidelines.xhtml

and d is the tolerable error of 0.05. Professional literature indicates that the minimum sample size for testing an mediator model is 200.[45] Taking into account a 20% sample loss rate, a total of 293 samples in this study would meet the minimum requirement.

Questionnaires with more than 10% missing values were excluded. IBM SPSS version 25 was used to complete the descriptive statistics and correlation analysis of all variables. If the data conformed to a normal distribution, the Pearson correlation coefficient was used to express the correlation between the two variables. The PROCESS macro in SPSS, developed by Hayes, was used for multiple mediation analyses. We adopted a bootstrap method (sampling was repeated 5000 times) to construct a 95% confidence interval for significance testing of mediating effects.

This study employed three regression models to investigate the role of helplessness and internality as parallel mediators in the relationship between health literacy/social support and self-management. Model 1 examined the influence of health literacy/social support on helplessness. Model 2 explored the impact of health literacy/social support on internality. Model 3 examined the combined effects of health literacy/social support, helplessness, and internality on self-management. Control variables such as age and gender were included as covariates in the models.

Patient and public involvement

Patients and/or the public were not involved in the design, conduct, reporting, or dissemination plans of this research.

For peer review only - http://bmjopen.bmj.com/site/about/guidelines.xhtml

RESULTS

Demographic characteristics

The study population consisted of 239 Chinese MHD patients aged 21-86 with a mean age of 55.61 and a standard deviation (SD) = 13.31. Among them, 59.8% were male. The characteristics of the participants are shown in Table 1.

Table 1 Descriptive statistics of sociodemographic characteristics (n=239).

Variable		n	%
Gender			
	Male	143	59.8
	Female	96	40.2
Age (years)			
	18-44	46	19.2
	45-59	100	41.8
	≥60	93	38.9
Education level			
	Elementary school	26	10.9
	Secondary school	126	52.7
	University and above	87	36.4
Employed status			
	On duty	10	4.2
	Part-time job	37	15.5
	Off duty	192	80.3
Protopathy			
	Hypertension	52	21.8
	Diabetes	48	20.1
	Chronic nephritis	105	43.9
	Polycystic kidney	20	8.4

For peer review only - http://bmjopen.bmj.com/site/about/guidelines.xhtml

For peer review only

	Others	14	5.9
Duration Of the MHD（years）			
	＜1	54	22.6
	1-3	42	17.6
	3-7	72	30.1
	≥ 7	71	29.7

Preliminary analysis

The correlations between the variables are listed in Table 2. First, social support, health literacy, and self-management were all negatively correlated with helplessness (r = -0.361, -0.599, and -0.463). Second, internality was positively associated with social support, health literacy, and self-management, with correlation coefficients of 0.445, 0.691, and 0.489, respectively. Third, social support and health literacy positively predicted self-management (r = 0.48, 0.636).

Table 2 Descriptive statistics and Pearson's correlation coefficients for all variables of interest (n=239).

Variable	M	SD	1	2	3	4	5
1.Helplessness	16.858	4.70	1				
2.Internality	17.351	6.411	-.140*	1			
3.Social Support	44.628	10.094	-.361**	.445**	1		
4.Self-Management	53.197	10.537	-.463**	.489**	.480**	1	
5.Health Literacy	16.889	3.921	-.599**	.691**	.443**	.636**	1

Note: *p＜0.05， ** p < 0.01. M=mean, SD=standard deviation.

For peer review only

Parallel multiple mediator models

This study used three regression models to examine the parallel mediators' role of helplessness and internality (Table 3 and Figure 1). The results of Model 1 showed that health literacy/social support was negatively associated with helplessness (β = -0.616, *P* < 0.001; β = -0.354, *P* < 0.001). In model 2, health literacy/social support was positively associated with internality (β = 0.666, *P* < 0.001; β = 0.416, *P* < 0.001). In Model 3, we found that in the health literacy model, the effects of both helplessness and internality on self-management were significant (β = -0.212, *P* < 0.01; β = 0.240, *P* < 0.01), and health literacy remained a significant positive predictor of self-management (β = 0.351, *P* < 0.001). Similarly, in the social support model, social support, helplessness, and internality all had significant effects on self-management (β = 0.199, *P* < 0.001; β = -0.331, *P* < 0.001; β = 0.376, *P* < 0.001). Therefore, we deduced that helplessness and internality partially mediate the relationship between health literacy/social support and self-management.

Table 3 Results of the multiple mediation analysis.

Regression Model		Fitting Indices		Regression Coefficient	
**Outcome Variable**	**Predictor Variable**	**R^2**	**F**	**β**	**t**
Helplessness	Social Support	0.402	6.357	-0.354	-5.808***
Internality	Social Support	0.523	12.422	0.416	7.337***
Self-management	Social Support	0.668	20.526	0.199	3.411***
	Helplessness			-0.331	-6.172***

For peer review only

For peer review only - http://bmjopen.bmj.com/site/about/guidelines.xhtml

	Internality			0.376	6.522***
Helplessness	Health Literacy	0.629	21.630	-0.616	-11.682***
Internality	Health Literacy	0.722	35.981	0.666	14.197***
	Health Literacy			0.351	3.635***
Self-management	Helplessness	0.671	20.828	-0.212	-2.998**
	Internality			0.240	3.028**

*Note: **p < 0.01, ***p < 0.001.*

To confirm the significance of the model's total, direct, and indirect effects, we used a bootstrap analysis with 5000 repeated sampling tests, and the confidence interval was set at 95%. The results showed significant parallel mediating effects of helplessness and internality on the relationship between health literacy/social support and self-management. The indirect effect size of health literacy via helplessness and internality on self-management was 0.780 (95% CI [0.373, 1.218]), accounting for 45.29% of the total effect. The indirect effect size of social support via helplessness and internality on self-management was 0.286 (95% CI [0.207, 0.377]), accounting for 57.88% of the total effect. In addition, the differences in effect sizes for helplessness and internality in the two models were -0.080 (95% CI [-0.374, 0.216]) and -0.041 (95% CI [-0.127, 0.043]), respectively. This result indicated that helplessness and internality did not differ significantly in the mediated effect size of the health literacy/social support and self-management relationship model. (Table 4)

Table 4 Bootstrap analysis of multiple mediation effects.

For peer review only

For peer review only - http://bmjopen.bmj.com/site/about/guidelines.xhtml

	Effect Size	SE	LLCI	ULCI
X1 (Social Support)				
Total	0.494	0.060	0.375	0.612
Direct	0.208	0.061	0.088	0.328
Total Indirect	0.286	0.043	0.207	0.377
Indirect effect (M1=Helplessness)	0.122	0.030	0.068	0.186
Indirect effect (M2=Internality)	0.163	0.032	0.108	0.230
(C1)	-0.041	0.043	-0.127	0.043
X2 (Health Literacy)				
Total	1.723	0.139	1.449	1.996
Direct	0.942	0.259	0.432	1.453
Total Indirect	0.780	0.217	0.373	1.218
Indirect effect (M1=Helplessness)	0.350	0.119	0.127	0.599
Indirect effect (M2=Internality)	0.430	0.143	0.161	0.720
(C1)	-0.080	0.148	-0.374	0.216

Note: C1=Helplessness minus Internality.

For peer review only

DISCUSSION

The findings confirm the COM-B model's applicability in health-related behavioral research among MHD patients. First, health literacy/social support, as an important competence and opportunity factor for MHD patients, was positively and negatively associated with internality and helplessness, respectively, and positively associated with self-management. Second, LH, an important motivational factor influencing individual behavior, and its two major systems, internality and helplessness, were

positively and negatively associated with self-management in MHD patients, respectively. Furthermore, our findings suggest that health literacy/social support influences self-management through internality and helplessness as mediating factors.

It is worth noting that internality, the core system of LH, has similarities with self-efficacy in that both can directly or indirectly influence self-management. However, the two are presented in different theoretical contexts, determining the intervention's core. Self-efficacy, derived from Bandura's social learning theory,[47] refers to an individual's subjective evaluation of his or her abilities, driven by personal expectations and goals. In this context, the core of self-management interventions is to help patients set reasonable goals and gradually develop their self-confidence. Internality originates from Rotter's locus of control theory,[31] which refers to a stable way of attributing the outcome of events internally after individuals have learned through experience. The core of its health behavior intervention is to guide patients to rational attribution, reduce helplessness and enhance motivation.

For peer review only

Correlation between health literacy/social support and LH

This study first verified the correlation between health literacy and the component of LH (internality/helplessness). According to the knowledge-attutide-practice theory,[48] patients with some knowledge of kidney disease and dialysis rated their self-behaviour better and had stronger beliefs about their illness. They could make rational judgments and perceptions when encountering negative disease-related events,

For peer review only - http://bmjopen.bmj.com/site/about/guidelines.xhtml

accompanied by a lower sense of helplessness, which was verified in this study. Conversely, patients with higher internality are more likely to acquire disease-related knowledge through various means, which is vital for patients' self-management. Those patients who were chronically helpless lacked the motivation to consistently learn self-care knowledge and skills. This point echoes the findings of our previous qualitative study.[49] Therefore, successful renal disease education should provide knowledge and increase motivation. Healthcare professionals should also emphasize patient internality and empowerment to help patients maintain a positive mindset and long-term intrinsic motivation to face their disease.

Next, this study verified the close relationship between internality/helplessness and social support. According to Shahriari,[50] social support, as an important external resource, can lead to significant psychological changes in patients. This study reflects two critical variables of internality and helplessness. On the one hand, a lack of social support can induce helplessness in patients, mainly depression, social avoidance, and abandonment. On the other hand, adequate social support can help patients expand access to social resources, increase internality in coping with illness, and reduce helplessness, which is similar to the findings of Wang[51] and Wu[52]. It is worth noting that Jansen[53] noted that excessive support given to MHD patients may affect their internality. In this regard, assisting patients to build an appropriate social support environment is essential to enhance their internality and temporarily escape from helplessness.

For peer review only - http://bmjopen.bmj.com/site/about/guidelines.xhtml

Relevance of LH to self-management

According to Seligman, LH is essential to human behavior.[54] The present study first verified that helplessness is a negative predictor of self-management behaviour, a result similar to other studies.[55-57] Helplessness plays an important cognitive, motivational, and behavioral role in individuals. It can influence patients' attitudes toward illness and treatment and determine their health-related behaviors and persistence.[58] MHD patients feel isolated due to worsening kidney function, complications, and death anxiety. In addition, anxiety, panic, depression, regret, and social isolation are all feelings that contribute to the emotional burden of MHD patients. Many accept their condition with a sense of helplessness—a feeling that whatever they do will not have an impact on the future. Overwhelming helplessness may weaken patients' motivation to engage in treatment. This sense of helplessness may also be reinforced by recurrent experiences of uncontrollable symptoms, which may be deeply rooted in the background of living with the situation.

Next, we verified that internality is a positive predictor of self-management, which is consistent with other studies.[59,60] Numerous scholars have argued that control is one of the basic psychological needs of humans, determined by humans' social attributes. Internality determines the degree to which an individual can grasp stressful events. When patients have high internality, they can actively use various resources and establish a more positive way of coping with their illness. In this study, MHD patients with higher internality tended to perceive the existing treatment outcome as a result of their behavior. For example, for adverse events in disease

For peer review only - http://bmjopen.bmj.com/site/about/guidelines.xhtml

treatment, patients more often attributed them to their lack of effort and believed they could mitigate the effects of adverse events by changing their behaviors.

The mediating role of LH

The results showed that health literacy/social support could influence the self-management of MHD patients through the mediating role of helplessness and internality, similar to other studies.[58,61] First, health literacy reflects patients' perceptions of illness and treatment and can determine their ability and motivation to take positive steps to improve their health. Patients with higher health literacy have a higher ability to access medical information and communicate with medical staff and are better able to make rational judgments independently when faced with changes in their health status, thereby reducing feelings of helplessness. Individuals with a correct understanding of the disease can influence their emotional responses. When MHD patients have more knowledge about kidney disease and health care, their ability to perceive disease deterioration is enhanced, and their sense of control over the disease increases. Based on this, patients can more actively and purposefully engage in their health behavior.

Social support plays a crucial role as an external resource, helping to buffer the effects of stressors. MHD patients often face challenges in disease control, the absence of social support, can contribute to feelings of helplessness.[62] Helplessness can lead patients to believe that they have limited control over their situation, attributing outcomes to external factors like fate, social background, or external forces.

For peer review only - http://bmjopen.bmj.com/site/about/guidelines.xhtml

This external locus of control reduces their initiative and leads to self-abandonment, social avoidance, and decreased trust in others. On the other hand, internality, combined with social support, creates a favorable internal and external environment for patients, providing them with resources and enhancing their confidence in controlling their disease outcome. This increased confidence in turn motivates patients to actively engage in health-related behaviors.

Implications for future research

The results confirmed that helplessness and internality are important ways for health literacy/social support to influence self-management. The results help us understand the mechanisms of possible changes in the health-related behaviors of MHD patients and provide theoretical support for future studies. In the future, health care providers can focus on LH as an intervention target and identify and assess the level of LH as early as possible while providing health education and medical support for MHD patients. Patients can be empowered by encouraging or rewarding their self-management behaviours to improve their internality and reduce helplessness.

Seligman et al.[63] showed that individual helplessness also triggers a group's helplessness, harming individual behavior. Most MHD patients in China have wardmate groups, and LH events among peers may impact other patients' behaviors. Future studies may investigate the interaction of LH in groups on MHD patients' behaviors.

For peer review only - http://bmjopen.bmj.com/site/about/guidelines.xhtml

Limitations and future directions

First, we selected dialysis patients from a southern city in China by convenience sampling, which may limit the generalizability of the results. Based on the ethnic diversity of China, future studies could recruit participants from more diverse cultures and backgrounds. Second, causality cannot be inferred from the current study, and future longitudinal studies are needed. Third, the reliance on self-reported data may pose a potential risk to the validity of the measurements.

CONCLUSION

This study sheds light on how health literacy and social support impact self-management in MHD patients. It emphasizes the role of LH in driving behavioral changes. The findings provide valuable insights for developing intervention strategies to enhance health-related behaviors among MHD patients. Clinical caregivers should prioritize preventing, identifying, and monitoring LH during health education and medical support.

Acknowledgements

We express our heartfelt gratitude to all members who assisted in the data collection and processing and to every MHD patient who participated in this study.

Contributors

For peer review only

CX and LL contributed to the conceptualization, formal analysis, and writing the original draft. CS, and YZ made contributions to the data curation and verification. YL and LZ contributed to the writing review and editing.

Funding

This research received grants from the National Natural Science Foundation of China (No.81873806), Major Scientific and Technological Projects in Hunan Province (No.2020SK2085), 2022 Hunan Province Postgraduate Scientific Research Innovation Project (No.12300-150110021), and 2022 Central South University Postgraduate Independent Exploration and Innovation Program (No. 1053320213471).

Competing interests

All authors declare that they have no conflicts of interest.

Patient consent for publication

Obtained.

Provenance and peer review

Not commissioned; externally peer reviewed.

Data availability statement

For peer review only

The datasets analysed during the current study are not publicly available because they contain information that could compromise research participant consent but are available from the corresponding author on reasonable request.

Figure legends

Figure 1. Parallel multiple mediator models. A: Health Literacy Model, B: Social Support Model.

Supplemental Figure. Theoretical framework based on the COM-B model. HL: Health literacy; LH: Learned helplessness; SS: Social support; SM: Self-management.

Ethics approval

The study was approved by the Ethics Committee of Xiangya Nursing School of Central South University on April 20, 2022 (No. E202255). Informed consent was obtained from all participants.

References

1 Collaboration GCKD. Global, regional, and national burden of chronic kidney disease, 1990–2017: a systematic analysis for the Global Burden of Disease Study 2017. *Lancet* 2020;395(10225):709-33. doi: 10.1016/S0140-6736(20)30045-3

2 O'Hare AM. Dialysis adequacy reconsidered: The person comes first. *Seminars in dialysis* 2020;33(6):486-89. doi: 10.1111/sdi.12938 [published Online First:

For peer review only - http://bmjopen.bmj.com/site/about/guidelines.xhtml

2020/11/27]

3 Wang S-Y, Zang X-Y, Fu S-H*, et al.* Factors related to fatigue in Chinese patients with end-stage renal disease receiving maintenance hemodialysis: a multi-center cross-sectional study. *Renal failure* 2016;38(3):442-50. doi: 10.3109/0886022X.2016.1138819

4 He Y, Lu Y, Yang S*, et al.* Dietary Plant Protein and Mortality Among Patients Receiving Maintenance Hemodialysis: A Cohort Study. *American journal of kidney diseases : the official journal of the National Kidney Foundation* 2021;78(5):649-57.e1. doi: 10.1053/j.ajkd.2021.03.023 [published Online First: 2021/05/31]

5 Meng Y, Wu H-t, Niu J-l*, et al.* Prevalence of depression and anxiety and their predictors among patients undergoing maintenance hemodialysis in Northern China: a cross-sectional study. *Ren Fail* 2022;44(1):933-44. doi: 10.1080/0886022X.2022.2077761

6 Brioni E, Delli Zotti G, Apuzzo L*, et al.* Self-efficacy and self-management in patients in hemodialysis: a narrative review of multidisciplinary theories. *G Ital Nefrol* 2021;38(3):2021-vol3. Italian.

7 Griva K, Nandakumar M, Jo-an HN*, et al.* Hemodialysis self-management intervention randomized trial (HED-SMART): a practical low-intensity intervention to improve adherence and clinical markers in patients receiving hemodialysis. *American Journal of Kidney Diseases* 2018;71(3):371-81. doi: 10.1053/j.ajkd.2017.09.014

For peer review only - http://bmjopen.bmj.com/site/about/guidelines.xhtml

8 Curtin RB, Sitter DC, Schatell D, *et al.* Self-management, knowledge, and functioning and well-being of patients on hemodialysis. *Nephrology Nursing Journal* 2004;31(4):378-86.

9 Tovazzi ME, Mazzoni V. Personal paths of fluid restriction in patients on hemodialysis. *Nephrology Nursing Journal* 2012;39(3):207-15.

10 Li H, Cao YD, Jiang YF, *et al.* Reliability and validity tests of Hemodialysis Self-Management Instrument (in Chinese). *Chinese Journal of Nursing* 2015;50(11):1392-95. doi: 10.3761/j.issn.0254-1769.2015.11.024

11 Joboshi H, Oka M. Effectiveness of an educational intervention (the Encourage Autonomous Self-Enrichment Program) in patients with chronic kidney disease: a randomized controlled trial. *International journal of nursing studies* 2017;67:51-58. doi: 10.1016/j.ijnurstu.2016.11.008

12 Nutbeam D. The evolving concept of health literacy. *Social science medicine* 2008;67(12):2072-78. doi: 10.1016/j.socscimed.2008.09.050

13 Lopez-Vargas PA, Tong A, Howell M, *et al.* Educational interventions for patients with CKD: a systematic review. *American Journal of Kidney Diseases* 2016;68(3):353-70. doi: 10.1053/j.ajkd.2016.01.022

14 Devraj R, Borrego M, Vilay AM, *et al.* Relationship between health literacy and kidney function. *Nephrology* 2015;20(5):360-67. doi: 10.1111/nep.12425

15 Wu SFV, Hsieh NC, Lin LJ, *et al.* Prediction of self-care behaviour on the basis of knowledge about chronic kidney disease using self-efficacy as a mediator. *Journal of clinical nursing* 2016;25(17-18):2609-18. doi: 10.1111/jocn.13305

For peer review only - http://bmjopen.bmj.com/site/about/guidelines.xhtml

16 Hu C, Lim P, Chang T. The effectiveness of case management on self-care and quality of life in chronic kidney disease patients. *The Journal of Health Science* 2012;14(1):13-28.

17 Tifferet S. Gender differences in social support on social network sites: A meta-analysis. *Cyberpsychology, behavior, social networking* 2020;23(4):199-209. doi: 10.1089/cyber.2019.0516

18 Cullen FT. Social support as an organizing concept for criminology: Presidential address to the Academy of Criminal Justice Sciences. *Justice Quarterly* 1994;11(4):527-59. doi: Cullen FT. Social support as an organizing concept for criminology: Presidential address to the Academy of Criminal Justice Sciences. Justice Quarterly 1994;11(4):527-59.

19 Cardol CK, Boslooper-Meulenbelt K, van Middendorp H*, et al.* Psychosocial barriers and facilitators for adherence to a healthy lifestyle among patients with chronic kidney disease: A focus group study. *BMC nephrology* 2022;23(1):1-16. doi: 10.1186/s12882-022-02837-0

20 Neri L, Martini A, Andreucci VE*, et al.* Regimen complexity and prescription adherence in dialysis patients. *American journal of nephrology* 2011;34(1):71-76. doi: 10.1159/000328391

21 Kauric-Klein Z. Predictors of nonadherence with blood pressure regimens in hemodialysis. *Patient preference adherence* 2013;7:973. doi: 10.2147/PPA.S45369

22 Song Y-y, Chen L, Wang W-x*, et al.* Social Support, Sense of Coherence, and

For peer review only - http://bmjopen.bmj.com/site/about/guidelines.xhtml

Self-Management among Hemodialysis Patients. *Western Journal of Nursing Research* 2022;44(4):367-74. doi: 10.1177/0193945921996648

23 Been-Dahmen JM, Grijpma JW, Ista E*, et al.* Self-management challenges and support needs among kidney transplant recipients: A qualitative study. *Journal of advanced nursing* 2018;74(10):2393-405. doi: 10.1111/jan.13730

24 Seligman ME. Learned helplessness as a model of depression. Comment and integration. *Journal of abnormal psychology* 1978;87(1):165-79. doi: 10.1037//0021-843x.87.1.49

25 Camacho EM, Verstappen SM, Chipping J*, et al.* Learned helplessness predicts functional disability, pain and fatigue in patients with recent-onset inflammatory polyarthritis. *Rheumatology (Oxford, England)* 2013;52(7):1233-8. doi: 10.1093/rheumatology/kes434 [published Online First: 2013/02/21]

26 Crielaard L, Nicolaou M, Sawyer A*, et al.* Understanding the impact of exposure to adverse socioeconomic conditions on chronic stress from a complexity science perspective. *BMC medicine* 2021;19(1):242. doi: 10.1186/s12916-021-02106-1 [published Online First: 2021/10/13]

27 Peterson C, Seligman ME, Vaillant GE. Pessimistic explanatory style is a risk factor for physical illness: a thirty-five-year longitudinal study. *Journal of personality and social psychology* 1988;55(1):23-7. doi: 10.1037//0022-3514.55.1.23 [published Online First: 1988/07/01]

28 Yu-Ying C, Li-Ya T. Applying Swanson's Caring Theory to a Patient With End

For peer review only - http://bmjopen.bmj.com/site/about/guidelines.xhtml

Renal Stage Disease Experiencing Hopelessness. *Journal of Nursing* 2021;68(2):99-106. doi: 10.6224/JN.202104_68(2).13

29 Kurita N, Wakita T, Fujimoto S*, et al.* Hopelessness and Depression Predict Sarcopenia in Advanced CKD and Dialysis: A Multicenter Cohort Study. *The journal of nutrition, health & aging* 2021;25(5):593-99. doi: 10.1007/s12603-020-1556-4 [published Online First: 2021/05/06]

30 DeVellis R, Callahan L. A brief measure of helplessness in rheumatic disease: the helplessness subscale of the Rheumatology Attitudes Index. *The Journal of rheumatology* 1993;20(5):866-69.

31 Rotter JB. Generalized expectancies for internal versus external control of reinforcement. *Psychol Monogr Gen Appl* 2010;80:1–28.

32 Khalil AA, Abed MA. Perceived social support is a partial mediator of the relationship between depressive symptoms and quality of life in patients receiving hemodialysis. *Archives of Psychiatric Nursing* 2014;28(2):114-18. doi: 10.1016/j.apnu.2013.11.007

33 Bayuo J, Bristowe K, Harding R*, et al.* "Managing uncertainty": Experiences of family members of burn patients from injury occurrence to the end-of-life period. *Burns : journal of the International Society for Burn Injuries* 2021;47(7):1647-55. doi: 10.1016/j.burns.2021.02.009 [published Online First: 2021/04/10]

34 Smallheer BA, Vollman M, Dietrich MS. Learned helplessness and depressive symptoms following myocardial infarction. *Clinical Nursing Research*

For peer review only - http://bmjopen.bmj.com/site/about/guidelines.xhtml

2018;27(5):597-616. doi: 10.1177/1054773816689752
35 Kail BL, Carr DC. Successful aging in the context of the disablement process: Working and volunteering as moderators on the association between chronic conditions and subsequent functional limitations. *The Journals of Gerontology: Series B* 2017;72(2):340-50. doi: 10.1093/geronb/gbw060
36 Michie S, Van Stralen MM, West R. The behaviour change wheel: a new method for characterising and designing behaviour change interventions. *Implementation science* 2011;6(1):1-12. doi: 10.1186/1748-5908-6-42
37 Murtagh EM, Barnes AT, McMullen J*, et al.* Mothers and teenage daughters walking to health: using the behaviour change wheel to develop an intervention to improve adolescent girls' physical activity. *Public health* 2018;158:37-46. doi: 10.1016/j.puhe.2018.01.012
38 Li H. Research on the Condition and Influencing Factors ofHemodialysis Patients' Self Management (in Chinese). Peking Union Medical College, 2011.
39 Xiao SY. The theoretical basis and research application of the Social Support Rating Scale (in Chinese). *Journal of Clinical Psychiatry* 1994;99(04):98–100. doi: CNKI:SUN:LCJS.0.1994-02-019
40 Jiang H, Wang L, Zhang Q*, et al.* Family functioning, marital satisfaction and social support in hemodialysis patients and their spouses. *Stress Health* 2015;31(2):166-74. doi: 10.1002/smi.2541
41 Song YJ. Construction and testing of a self-management scale for hemodialysis patients (in Chinese). Kaohsiung Medical University, 2009.

For peer review only - http://bmjopen.bmj.com/site/about/guidelines.xhtml

42 Li H, Cao YD, Jiang YF, *et al.* Introduction and reliability testing of a self-management scale for hemodialysis patients (in Chinese). *Chin J Nurs* 2015;50(11):1392-95. doi: 10.3761/j.issn.0254-1769.2015.11.024

43 Nicassio PM, Wallston KA, Callahan LF, *et al.* The measurement of helplessness in rheumatoid arthritis. The development of the arthritis helplessness index. *J Rheumatol* 1985;12(3):462-67. doi: 10.1007/BF02031627

44 Xie C, Zhou L, Sun C, *et al.* The cross-cultural adaptation and psychometric evaluation of a learned helplessness scale for maintenance hemodialysis patients in China (LHS-MHD-C). *Res Nurs Health* 2023;Advance online publication doi: 3.80599.net/10.1002/nur.22298

45 Wu ML. Structural equation model: the operation and application of AMOS. Chongqing: Chongqing University Press 2010.

46 Charan J, Biswas T. How to calculate sample size for different study designs in medical research? *Indian J Psychol Med* 2013;35(2):121-6. doi: 10.4103/0253-7176.116232 [published Online First: 2013/09/21]

47 Bandura A. The explanatory and predictive scope of self-efficacy theory. *J Soc Clin Psychol* 1986;4(3):359-73. doi: 10.1521/jscp.1986.4.3.359

48 Oshima T, Siah KT, Kim YS, *et al.* Knowledge, attitude, and practice survey of gastroparesis in Asia by Asian Neurogastroenterology and Motility Association. *Journal of neurogastroenterology motility* 2021;27(1):46. doi: 10.5056/jnm20117

49 Xie C, Li L, Li Y. "Alive Day is the Day": A Qualitative Study of Experiences of

For peer review only - http://bmjopen.bmj.com/site/about/guidelines.xhtml

Learned Helplessness in Maintenance Haemodialysis Patients. *Risk Manag Healthc Policy* 2023:231-45. doi: 10.2147/RMHP.S401205

50 Shahriari M, Alimohammadi N, Ahmadi M. Effects of a family centered program on perceived social support in patients with congestive heart failure: An interventional study. *Journal of Education Health Promotion International* 2016(Vol. 5):1-6. doi: 10.4103/2277-9531.184559

51 Wang XN, Zhang H, Xie ZY. Analysis of the current status of self-concealment in maintenance hemodialysis patients and its correlation and influencing factors with disease stigma and social support (in Chinese). *Shandong Medical Journal* 2020;60(17):84-86. doi: 10. 3969 / j. issn. 1002-266X. 2020. 17. 025

52 Wu H. Self-perceived burden and uncertainty in illness in maintenance hemodialysis patients and related research on social support (in Chinese). Southwest Medical University, 2020.

53 Jansen DL, Rijken M, Kaptein AA*, et al.* The role of social support in dialysis patients' feelings of autonomy and self-esteem: Is support more beneficial for patients with specific illness perceptions? *Families, Systems, Health* 2014;32(3):313. doi: 10.1037/fsh0000028

54 Seligman M, Weiss J, Weinraub M*, et al.* Coping behavior: learned helplessness, physiological change and learned inactivity. *Behaviour research therapy* 1980;18(5):459-512. doi: 10.1016/0005-7967(80)90011-x

55 Neuvonen E, Lehtisalo J, Solomon A*, et al.* Psychosocial determinants for adherence to a healthy lifestyle and intervention participation in the FINGER

For peer review only - http://bmjopen.bmj.com/site/about/guidelines.xhtml

trial: an exploratory analysis of a randomised clinical trial. *Aging clinical and experimental research* 2022;34(8):1793-805. doi: 10.1007/s40520-022-02088-x [published Online First: 2022/02/20]

56 Sheridan N, Kenealy T, Salmon E*, et al.* Helplessness, self blame and faith may impact on self management in COPD: a qualitative study. *Primary Care Respiratory Journal* 2011;20(3):307-14. doi: 10.4104/pcrj.2011.00035

57 Omachi TA, Katz PP, Yelin EH*, et al.* The COPD Helplessness Index: a new tool to measure factors affecting patient self-management. *Chest* 2010;137(4):823-30. doi: 10.1378/chest.09-0764 [published Online First: 2009/10/20]

58 Wang LM, Chiou CP. Effectiveness of interactive multimedia CD on self-care and powerlessness in hemodialysis patients. *Journal of Nursing Research* 2011;19(2):102-11. doi: 10.1097/JNR.0b013e31821b0eff

59 Fan JL, Kong Y, Shi SH*, et al.* Correlation Study of Health Locus of Control and Self-management Behaviors in Patients Underwent Hemodialysis (in Chinese). *Nursing Journal of Chinese People's Liberation Army* 2016;33(9):23-27. doi: 10.3969/j.issn.1008-9993.2016.09.005

60 Lael-Monfared E, Tehrani H, Teiho Z*, et al.* The study of eye care behaviors in patients with type 2 diabetes. *Journal of Diabetes Metabolic Disorders* 2020;19(1):257-63. doi: 10.1007/s40200-020-00499-z

61 Chen CC, Li HP, Liu HQ*, et al.* A study on the relationship among self - management behavior, family function, locus of control among maintenance

For peer review only

hemodialysis patient (in Chinese). *Chinese Journal of health education* 2020;36(11):1053. doi: 10.16168/j.cnki.issn.1002-9982.2020.11.020

62 Kiajamali M, Hosseini M, Estebsari F*, et al.* Correlation between social support, self-efficacy and health-promoting behavior in hemodialysis patients hospitalized in Karaj in 2015. *Electronic Physician* 2017;9(7):4820. doi: 10.19082/4820

63 Abramson LY, Seligman ME, Teasdale JD. Learned helplessness in humans: critique and reformulation. *Journal of abnormal psychology* 1978;87(1):49. doi: 10.1037//0021-843x.87.1.49

For peer review only - http://bmjopen.bmj.com/site/about/guidelines.xhtml

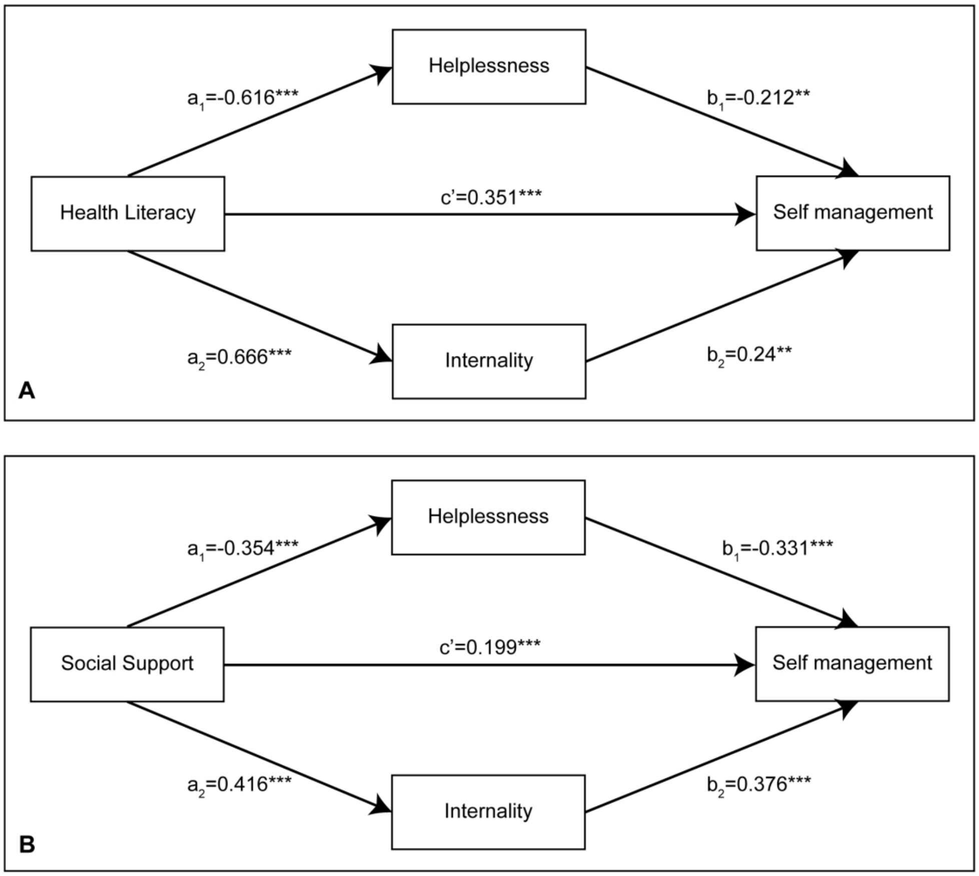

Parallel multiple mediator models. A: Health Literacy Model, B: Social Support Model.

207x185mm (300 x 300 DPI)

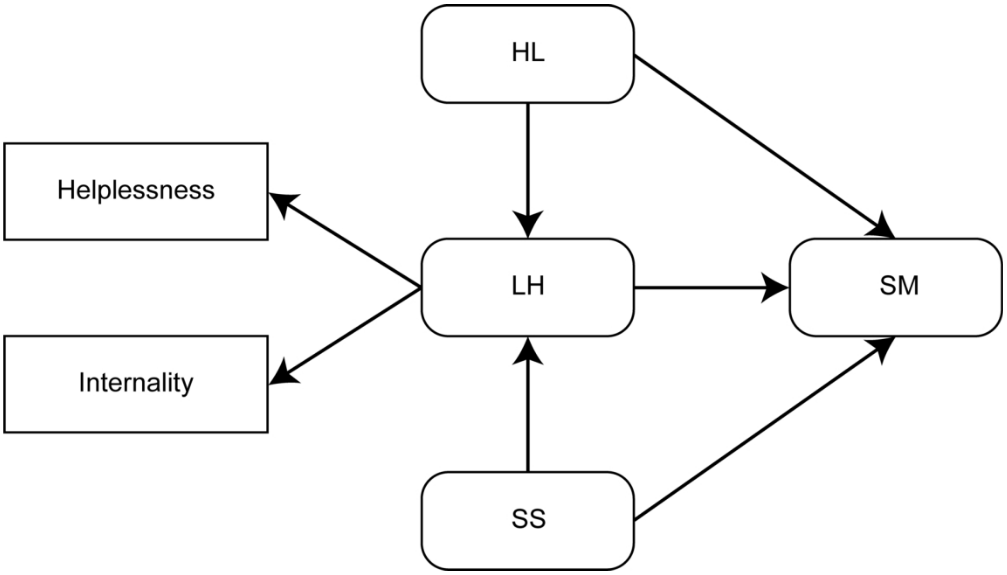

159x90mm (300 x 300 DPI)

Supplementary File 1

STROBE 2007 (v4) Statement—Checklist of items that should be included in reports of *cross-sectional studies*

**Section/Topic**	**Item #**	**Recommendation**	**Reported on page #**
**Title and abstract**	1	(*a*) Indicate the study's design with a commonly used term in the title or the abstract	P1
		(*b*) Provide in the abstract an informative and balanced summary of what was done and what was found	P1-2
**Introduction**			
Background/rationale	2	Explain the scientific background and rationale for the investigation being reported	P3-7
Objectives	3	State specific objectives, including any prespecified hypotheses	P8
**Methods**			
Study design	4	Present key elements of study design early in the paper	P8
Setting	5	Describe the setting, locations, and relevant dates, including periods of recruitment, exposure, follow-up, and data collection	P8-9
Participants	6	(*a*) Give the eligibility criteria, and the sources and methods of selection of participants	P8-9
Variables	7	Clearly define all outcomes, exposures, predictors, potential confounders, and effect modifiers. Give diagnostic criteria, if applicable	P9-11
Data sources/ measurement	8*	For each variable of interest, give sources of data and details of methods of assessment (measurement). Describe comparability of assessment methods if there is more than one group	P9-11
Bias	9	Describe any efforts to address potential sources of bias	P11
Study size	10	Explain how the study size was arrived at	P11
Quantitative variables	11	Explain how quantitative variables were handled in the analyses. If applicable, describe which groupings were chosen and why	P11
Statistical methods	12	(*a*) Describe all statistical methods, including those used to control for confounding	P13
		(*b*) Describe any methods used to examine subgroups and interactions	P12-13
		(*c*) Explain how missing data were addressed	P12
		(*d*) If applicable, describe analytical methods taking account of sampling strategy	P9
		(*e*) Describe any sensitivity analyses	N/A

For peer review only

**Results**			
Participants	13*	(a) Report numbers of individuals at each stage of study—eg numbers potentially eligible, examined for eligibility, confirmed eligible, included in the study, completing follow-up, and analysed	N/A
		(b) Give reasons for non-participation at each stage	N/A
		(c) Consider use of a flow diagram	N/A
Descriptive data	14*	(a) Give characteristics of study participants (eg demographic, clinical, social) and information on exposures and potential confounders	P13-14
		(b) Indicate number of participants with missing data for each variable of interest	N/A
Outcome data	15*	Report numbers of outcome events or summary measures	P15
Main results	16	(*a*) Give unadjusted estimates and, if applicable, confounder-adjusted estimates and their precision (eg, 95% confidence interval). Make clear which confounders were adjusted for and why they were included	P13
		(*b*) Report category boundaries when continuous variables were categorized	N/A
		(*c*) If relevant, consider translating estimates of relative risk into absolute risk for a meaningful time period	N/A
Other analyses	17	Report other analyses done—eg analyses of subgroups and interactions, and sensitivity analyses	N/A
**Discussion**			
Key results	18	Summarise key results with reference to study objectives	P18
Limitations	19	Discuss limitations of the study, taking into account sources of potential bias or imprecision. Discuss both direction and magnitude of any potential bias	P23
Interpretation	20	Give a cautious overall interpretation of results considering objectives, limitations, multiplicity of analyses, results from similar studies, and other relevant evidence	P18-22
Generalisability	21	Discuss the generalisability (external validity) of the study results	P23
**Other information**			
Funding	22	Give the source of funding and the role of the funders for the present study and, if applicable, for the original study on which the present article is based	P24

*Give information separately for cases and controls in case-control studies and, if applicable, for exposed and unexposed groups in cohort and cross-sectional studies.

Note: An Explanation and Elaboration article discusses each checklist item and gives methodological background and published examples of transparent reporting. The STROBE checklist is best used in conjunction with this article (freely available on the Web sites of PLoS Medicine at http://www.plosmedicine.org/, Annals of Internal Medicine at http://www.annals.org/, and Epidemiology at http://www.epidem.com/). Information on the STROBE Initiative is available at www.strobe-statement.org.

For peer review only
